# MSC-encapsulated porous microparticle eye drops for autoimmune dry eye disease treatment in NOD mice

**DOI:** 10.1126/sciadv.adu9772

**Published:** 2025-09-24

**Authors:** Taige Chen, Rui Liu, Qin Chen, Xuebing Feng, Bin Kong, Yuanjin Zhao

**Affiliations:** ^1^ Department of Rheumatology and Immunology, Nanjing Drum Tower Hospital, Affiliated Hospital of Medical School, Nanjing University, Nanjing 210008, China.; ^2^ Department of Biomedical Engineering, School of Medicine, Shenzhen University, Shenzhen, Guangdong 518000, China.

## Abstract

Dry eye disease (DED) is characterized by chronic inflammation and an unstable tear film. Stem cells have shown potential for DED treatment, but the main challenge lies in improving cell delivery effectiveness. Here, we developed eye drops for autoimmune DED treatment using porous arginine–glycine–aspartic acid (RGD)–modified alginate microcarriers with mesenchymal stem/stromal cells (MSCs) (RGD-Alg@MSCs). These microcarriers provided a favorable microenvironment for large-scale cell expansion while maintaining stemness with ideal mechanical properties for ocular application. In vitro, RGD-Alg@MSCs demonstrated significantly enhanced therapeutic effects compared to conventional MSCs, including improved cell viability, reduced apoptosis and reactive oxygen species, and enhanced release of immunomodulatory factors. Transcriptomic analysis revealed distinct molecular mechanisms underlying these enhanced therapeutic effects. In the mouse model, RGD-Alg@MSCs exhibited prolonged ocular retention and enhanced tear production, promoted corneal healing, and suppressed inflammation by inhibiting dendritic cell activation and T_H_17 differentiation. Our microcarrier system substantially improves stem cell delivery efficiency for treating autoimmune DED.

## Teaser

MSC-loaded porous microcarriers improve retention and immune regulation for treating autoimmune dry eye disease.

## INTRODUCTION

Dry eye disease (DED) is a prevalent ocular disease characterized by an unstable tear film and persistent inflammation, which cause discomfort, visual impairment, and damage to the surface of the eye. DED not only involves a lack of tears but also an immune-mediated inflammatory response that affects both the innate and adaptive immune systems. Autoimmune-related DED, such as that associated with Sjögren’s syndrome (SS), represents a particularly challenging form of the disease with substantial inflammatory components. The global burden of autoimmune-related DED is considerable, with epidemiological studies revealing remarkably high prevalence rates across various autoimmune conditions. Notably, up to 95% of patients with SS experience dry eye symptoms, while notable rates are also observed in rheumatoid arthritis (38 to 47%), systemic lupus erythematosus (10 to 21%), and systemic sclerosis (37 to 79%) patients (*1*, *2*). This high prevalence translates into considerable impact on the life quality of patients, with many experiencing daily symptoms that markedly interfere with routine activities such as reading, driving, and digital screen use. Beyond the physical discomfort, autoimmune-related DED imposes a profound psychological burden, with anxiety and depression rates ranging from 26.5% to 83.8% among affected patients (*3*), representing a 37% higher risk compared to conventional dry eye patients (*4*). Moreover, the economic burden is equally concerning, encompassing both direct medical costs for treatments and substantial indirect costs from reduced workplace productivity and absenteeism. For example, a study on patients with SS in East China found that their annual total expenditure on treatment was approximately 5.5 times higher than that of patients with non-Sjögren’s dry eye (*4*). These epidemiological insights underscore the urgent clinical need for more effective therapeutic interventions for autoimmune-related DED. Existing therapies, including artificial tears, anti-inflammatory medications, and surgical procedures, frequently offer only short-term alleviation of symptom and cannot effectively target the fundamental immunological malfunction. Notably, stem cells are a potential treatment for autoimmune DED because they can control immune responses, decrease inflammation, and facilitate tissue repair and regeneration (*5*). Mesenchymal stem/stromal cells (MSCs) have the ability to heal the tissue, reduce proinflammatory cytokines, halt the activation of dendritic cells (DCs), and decrease T cell proliferation. As a result, an anti-inflammatory environment that encourages healing is created (*6*). Cell-based therapies represent a promising frontier in ophthalmology, offering potential for treating conditions resistant to conventional therapies. However, effective implementation faces several challenges, including limited cell expansion capability, poor survival in harsh environments, and inefficient delivery methods. Current approaches for MSC administration typically rely on intravenous injection, presenting considerable limitations for ocular therapy. When delivered systemically, only a small fraction of stem cells reach the eye, while direct ocular injection can be invasive and may compromise cell survival in the harsh ocular microenvironment. Additionally, the dynamic conditions of the ocular surface, including blinking and tear flow, rapidly clear conventional cell-based eye drops, limiting therapeutic effectiveness. Therefore, innovative delivery methods are urgently needed to enhance MSC retention, survival, and therapeutic efficacy for treating ocular conditions like autoimmune DED.

Herein, we propose using porous hydrogel microcarriers to deliver MSCs as eye drops for treating autoimmune DED. Hydrogels are a promising method for stem cell delivery due to their compatibility with living tissue, customizable nature, and ability to mimic the natural environment of extracellular matrix (*7*, *8*). Hydrogels can be modified and engineered to improve cell distribution (*9*). Nevertheless, conventional hydrogel systems often lack porous structures, which restricts nutrient diffusion, impedes therapeutic factor release, and limits interactions between encapsulated stem cells and their environment (*10*–*12*). The method of microfluidic electrospray makes it possible to produce consistent hydrogel microcarriers with exact control over their size and composition, overcoming the limitations of conventional systems and enhancing the effectiveness of stem cell treatments (*13*). These microcarriers create an ideal milieu for encapsulating MSCs, guaranteeing consistency and scalability in manufacturing (*14*). However, this method remains largely unexplored for DED and offers a groundbreaking approach with considerable potential to enhance the delivery of MSCs, providing an innovative solution for DED treatment.

**Fig. 1.**
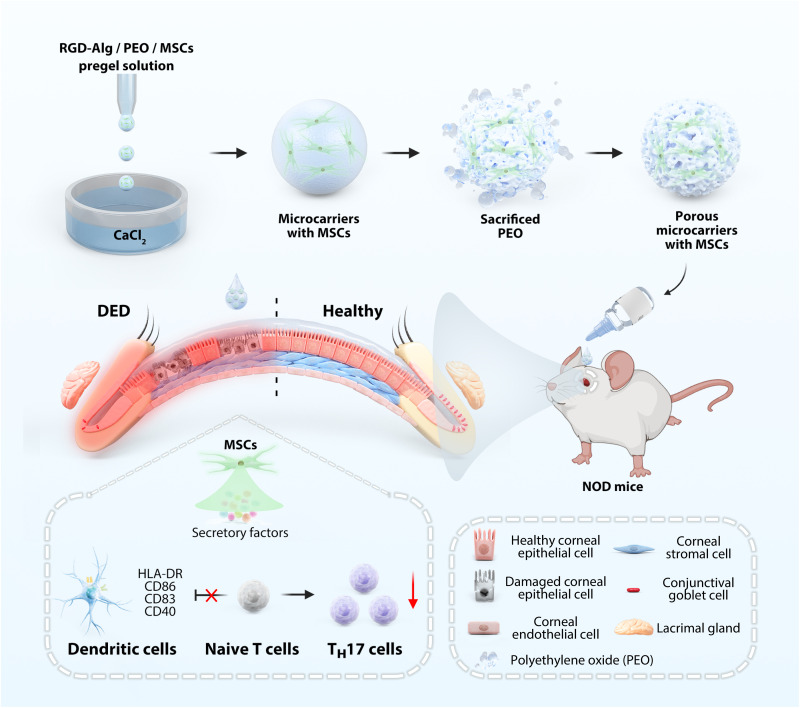
Schematic of RGD-Alg microcarrier fabrication and application. Schematic of the fabrication of the porous RGD-Alg microcarriers with MSC encapsulation and their application in promoting autoimmune DED in NOD mice.

Here, we fabricated porous microparticles encapsulating MSCs based on microfluidic electrospray for DED treatment (Fig. 1). The microcarriers were created by electrospraying the solution of arginine–glycine–aspartic acid (RGD)–modified sodium alginate with polyethylene oxide (PEO) and MSCs into the calcium chloride (CaCl_2_) solution. As PEO dissolved in water, it created channels within the microcarriers that facilitate material exchange, nutrient diffusion, and release of bioactive compounds from MSCs, resulting in persistent therapeutic advantages. These microcarriers incorporate RGD peptide modification to enhance cell attachment and stability, significantly improving the therapeutic potential of the encapsulated MSCs. Their mechanical properties balance structural integrity with softness, while their shear-thinning behavior resembles conventional eye drop solutions, ensuring ocular comfort and adaptability to the dynamic eye environment. Our stability studies demonstrated that RGD-Alg@MSCs maintained structural integrity and proper cell viability for at least 24 hours when stored at 4°C, showing superior stability compared to MSCs in direct suspension. The porous microcarriers were evaluated in vitro using a hyperosmotic corneal epithelial cell culture that replicates dry eye circumstances. The results demonstrated notable enhancements in cell viability, reductions in apoptosis and reactive oxygen species (ROS), and diminished expression of proinflammatory cytokines. RGD-Alg@MSCs showed significantly superior protective effects for apoptosis and enhanced secretory factors compared to conventional MSCs. Additionally, transcriptomic analysis through RNA sequencing (RNA-seq) revealed distinct molecular mechanisms underlying the enhanced therapeutic effects of microcarrier-encapsulated MSCs. In the in vivo study, comprehensive biocompatibility assessments confirmed the safety of RGD-Alg@MSCs, with terminal deoxynucleotidyl transferase (TdT)–mediated deoxyuridine triphosphate (dUTP) nick end labeling (TUNEL) staining revealing no significant apoptosis in ocular tissues following treatment. In vivo imaging system demonstrated that RGD-Alg@MSCs exhibited substantially enhanced retention at the ocular surface. An autoimmune-related DED model was established using male nonobese diabetic (NOD) mice. The results demonstrated that MSC-loaded microcarriers significantly improved tear production, corneal healing, restoration of conjunctival goblet cells, and reduction of inflammation. Furthermore, the therapy led to a noteworthy reduction in DC activation and T helper 17 (T_H_17) cell differentiation in vivo and in vitro, both of which are critical elements in the development of autoimmune DED. Thus, our MSC-encapsulated porous microcarrier system showed great potential for improving stem cell delivery over traditional methods and providing a more effective, long-term therapeutic option for autoimmune DED.

## RESULTS

### Synthesis and characterization of RGD-alginate

**Fig. 2.**
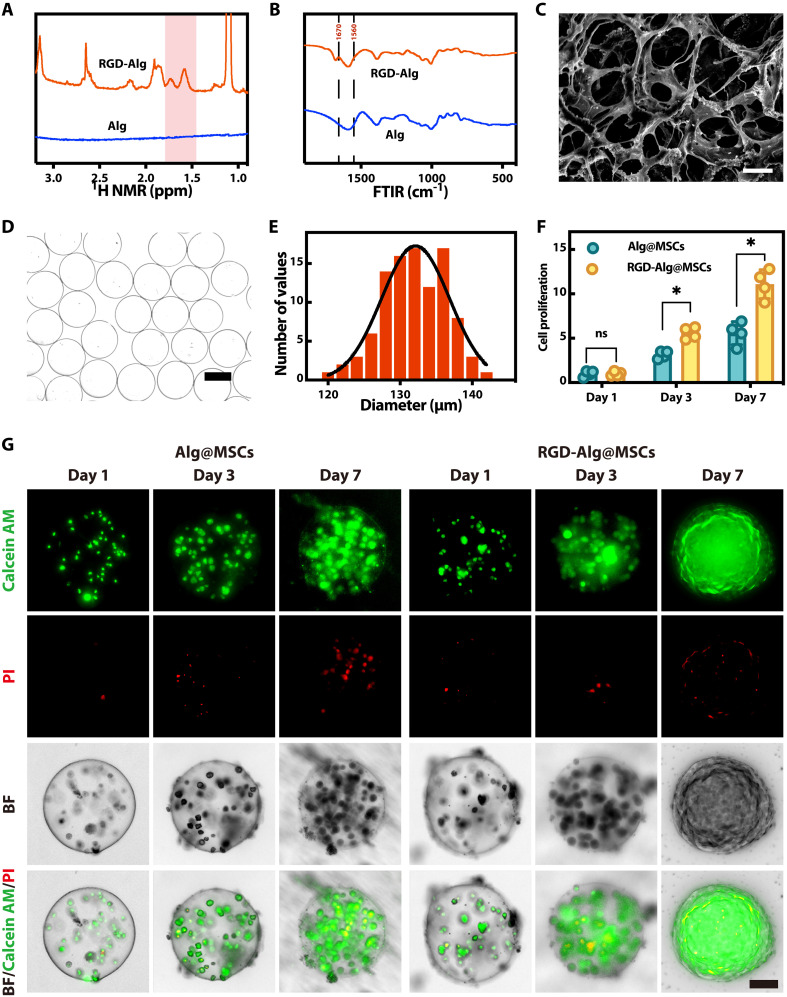
Characterization and analysis of RGD-Alg hydrogel and MSC-laden microcarriers. (**A**) NMR and (**B**) FTIR spectra of RGD-Alg hydrogel. (**C**) SEM picture of the porous RGD-Alg hydrogel. Scale bar, 10 μm. (**D**) Image of monodisperse RGD-Alg microparticles in the bright field. Scale bar, 100 μm. (**E**) Distribution of RGD-Alg microcarrier particle sizes. (**F**) Quantification of cell proliferation of the laden MSCs (*n* = 4). (**G**) Fluorescence images of MSCs loaded on days 1, 3, and 7 within the microcarriers. Calcein AM and PI were used for staining. Scale bar, 50 μm.

RGD-alginate (RGD-Alg) was synthesized by grafting RGD peptides onto the alginate with 1-ethyl-3-(3-dimethylaminopropyl) carbodiimide (EDC) and *N*-hydroxysuccinimide (NHS) coupling chemistry, following established protocols in the literature (*15*). This process ensures the effective grafting of RGD peptides, known for enhancing cell adhesion, to the alginate structure. The composite RGD-Alg was characterized using proton nuclear magnetic resonance spectroscopy (^1^H NMR) spectroscopy. The RGD-modified alginate showed proton peaks corresponding to arginine residues at 1.4 to 1.8 parts per million (ppm), indicating that the RGD peptide was successfully grafted onto the alginate (Fig. 2A and fig. S1). Further structural analysis was done using Fourier transform infrared (FTIR) (Fig. 2B) to ensure the successful alteration of RGD. The distinctive infrared absorption peaks of the alginate appeared at around 1400 and 1600 cm^−1^. New characteristic peaks were observed in the RGD-Alg samples at approximately 1670 and 1560 cm^−1^, corresponding to the amide I and amide II bands, respectively, which confirmed the successful formation of amide bonds between RGD peptides and alginate chains. The influence of RGD-Alg hydrogels on cell adhesion was analyzed and contrasted with unmodified alginate hydrogels (fig. S2). As expected, RGD-Alg hydrogel significantly promoted cell adhesion, demonstrating the effectiveness of RGD modification in enhancing the cell-friendly properties of the hydrogel (fig. S3). We adjusted the pore size by varying the PEO ratio. The final hydrogel precursor solution contained 2% RGD-Alg with PEO concentrations of 0.01%, 0.05%, 0.1%, 0.5%, 1%, 1.5%, and 2%. After crosslinking in 2% CaCl_2_ solution, the gel was transferred to phosphate-buffered saline (PBS) for 5 min to dissolve the PEO and form pores. Scanning electron microscopy (SEM) images showed the microstructure and quantified pore sizes. As PEO increased, pore size grew from 1 to 15 μm, forming interconnected, honeycomb-like pores (fig. S4). Considering MSC size and efficient water and nutrient exchange, 0.1% PEO was selected as the final concentration for preparing porous hydrogel microcarriers.

### Preparation of porous RGD-Alg microcarriers

During the experiment, an RGD-Alg solution containing 0.1% PEO was precisely injected through a microfluidic device during the electrospray procedure. The combined pregel solution experienced surface tension and transformed into microdroplets due to the high-voltage electric field generated by a direct current apparatus. The droplets then polymerized following the cross-linking process upon immersion in the CaCl_2_ solution and were then soaked in PBS to eliminate the PEO. Using optical microscopy, we confirmed the morphological characteristics of the microcarriers. The optimal parameter was assessed by measuring the diameters of several microcarriers under diverse circumstances. The findings demonstrate that the particle size of microcarriers may be accurately regulated by modifying three critical parameters: the electric field voltage between the microfluidic outlet and the collecting buffer surface, the concentration of the RGD-Alg solution, and the flow velocity (fig. S5). By fine-tuning these variables, we identified optimal conditions for producing microcarriers that meet our design specifications. More specifically, porous RGD-Alg microcarriers with diameters in about 130 μm were produced using an electric field voltage of 8 kV, a 2% RGD-Alg solution, and a flow rate of 100 μl/min (Fig. 2, D and E). The microcarriers were successfully synthesized, and the mass, stable, and uniform synthesis of these microcarriers may be achieved using our microfluidic electrospray fabrication method.

### Mechanical and rheological properties of the porous RGD-Alg microcarriers

To characterize the mechanical properties of the porous RGD-Alg microcarriers, Young’s modulus was measured using a micrometer-scale mechanical testing system. The microcarriers exhibited a Young’s modulus of approximately 50 kPa at 25% deformation (fig. S6), indicating a soft and elastic nature suitable for ocular applications. Rheological properties of the porous RGD-Alg microcarriers were assessed and compared with common eye drop solutions including polyvinyl alcohol (PVA), sodium hyaluronate, and sodium carboxymethyl cellulose (CMC) (fig. S7). Steady shear tests were performed over a shear rate range of 350 to 2350 s^−1^, approaching the blink shear frequency of approximately 4250 s^−1^ or higher (*16*), within the measurement capabilities of the test machine. The porous RGD-Alg microcarriers exhibited a viscosity (η) decrease from 250 to 50 mPa·s, demonstrating a shear-thinning behavior. This aligns with the ideal viscosity range of 30 to 300 mPa·s for effective ophthalmic drug delivery, balancing drug release, comfort, and corneal residence time (*17*). At higher shear rates, the viscosity may further decrease, potentially nearing that of common eye drops. This shear-thinning property, combined with the soft elastic nature of the microcarriers, suggests reduced friction during blinking and reduced potential foreign body sensation.

### Biocompatibility of the porous RGD-Alg microcarriers

To assess the biocompatibility of the porous RGD-Alg microspheres, human corneal epithelial cells (HCECs) and MSCs were cultured normally as the control group, while cultured in a medium exposed to the extract from the porous microspheres as the experimental group. Over a 3-day culture period, both control and experimental groups exhibited high confluence and robust proliferation (fig. S8), with cell viability consistently above 95% (fig. S9), as determined by calcein AM/propidium iodide (PI) staining. Besides, the viability of encapsulated cells depends on the ability of RGD-Alg hydrogel to retain water, as demonstrated by their ability to achieve a swelling ratio of 1300 wt % after incubation in deionized water (fig. S10). MSCs were loaded into porous RGD-Alg microcarriers (RGD-Alg@MSCs) by mixing them into the pregel solution. Further testing was conducted using calcein AM/PI staining to evaluate the cell viability and cell proliferation of the MSCs encapsulated in the microcarriers over a 7-day culture period. The RGD-Alg group exhibited a similar cell viability as did the porous alginate microcarrier control group (fig. S11) and a significantly higher cell proliferation than did the Alg group (Fig. 2G), indicating a superior biocompatible environment provided by the porous RGD-Alg microcarriers. The quantitative fluorescence data (Fig. 2F) corroborated this, highlighting the pro-proliferative effect of the RGD modification and the porous structure of the microcarriers. Beyond cell viability, maintaining cell stemness is crucial for therapeutic efficacy. Cell stemness is essential for efficient tissue regeneration and repair. To evaluate the stemness of MSCs cultured in the microcarriers, flow cytometry was performed after a 7-day coculture period. The expression levels of surface marker proteins in MSCs released from the microcarriers were statistically comparable to those of MSCs cultured on traditional tissue culture plates (Figs. S12 and S13). This result confirmed that the RGD-Alg microcarriers can effectively maintain the stemness of MSCs, preserving their therapeutic potential (*18*).

### Stability of RGD-Alg@MSCs

To evaluate the potential for clinical application, we assessed the stability of RGD-Alg@MSC eye drops during storage at 4°C in PBS. The microcarriers maintained their spherical morphology over 24 hours with limited degradation (fig. S14). Flow cytometry analysis revealed that MSCs encapsulated in the porous RGD-Alg microcarriers retained higher viability (83.6%) after 24 hours of cold storage compared to conventional suspended MSCs in PBS (69.3%), demonstrating the protective effect of the microcarrier system (fig. S15).

For clinical application, RGD-Alg@MSC eye drops are packaged in sterile dropper bottles under aseptic conditions and stored at 4°C to preserve microcarrier integrity and MSC bioactivity (fig. S16). Patients should gently shake the bottle before use to ensure uniform microcarrier distribution and then apply several drops as directed. This protocol maintains therapeutic efficacy while ensuring convenient administration.

### In vitro effect of RGD-Alg@MSCs

**Fig. 3.**
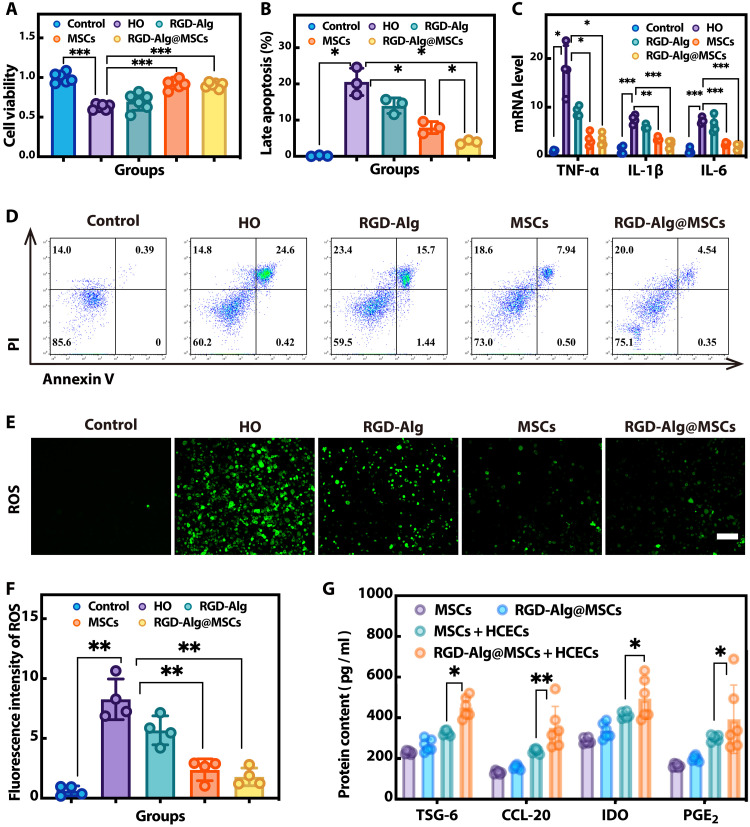
RGD-Alg@MSCs exhibited an in vitro protective effect. (**A**) A CCK-8 kit was used to evaluate HCEC viability under hyperosmotic stress (*n* = 6). (**B** and **D**) Flow cytometry was used to determine the late apoptosis rate (*n* = 3). (**C**) mRNA levels of typical inflammatory factors in the HCECs (*n* = 4). (**E**) Fluorescence microscopy images of ROS in HCECs. (**F**) Quantification of ROS fluorescence intensity (*n* = 4). (**G**) Levels of factors released measured by ELISA (*n* = 6). HO, hyperosmolarity.

The in vitro hypertonic model simulates the critical process of tear film instability (*19*), which is the central feature of DED. To assess how RGD-Alg@MSCs affected HCECs under hyperosmotic stress, we cultured HCECs in a hypertonic medium and cocultured them with RGD-Alg, MSCs, and RGD-Alg@MSCs. Cell viability assays demonstrated that both MSCs and RGD-Alg@MSCs significantly rescued the viability of HCECs in the hypertonic environment (Fig. 3A). Moreover, the rate of HCEC apoptosis was evaluated using flow cytometry. When compared to MSCs, the percentage of late apoptotic cells was significantly lower with RGD-Alg@MSCs (Fig. 3, B and D). Furthermore, the mRNA expression of tumor necrosis factor-α (TNF-α), interleukin-6 (IL-6), and IL-1β in HCECs is inhibited by RGD-Alg@MSCs (Fig. 3C), which indicated a strong anti-inflammatory effect, crucial for mitigating dry eye symptoms (*20*, *21*). The ROS level was assessed by fluorescence microscopy and the quantity of the fluorescence intensity (Fig. 3, E and F). RGD-Alg@MSCs demonstrated a significant reduction in ROS levels. ELISA assays further quantified the levels of typical cytokines in the coculture medium. The results showed that compared to MSCs, RGD-Alg@MSCs stably produced and released significantly higher levels of TNF-stimulated gene-6 (TSG-6), CC motif chemokine ligand 20 (CCL-20), indoleamine 2,3-dioxygenase (IDO), and prostaglandin E_2_ (PGE_2_) (Fig. 3G), which are vital for enhancing anti-inflammation and immune regulation and promoting tissue repair (*22*–*25*).

### Transcriptomic analysis of MSCs and HCECs in coculture

**Fig. 4.**
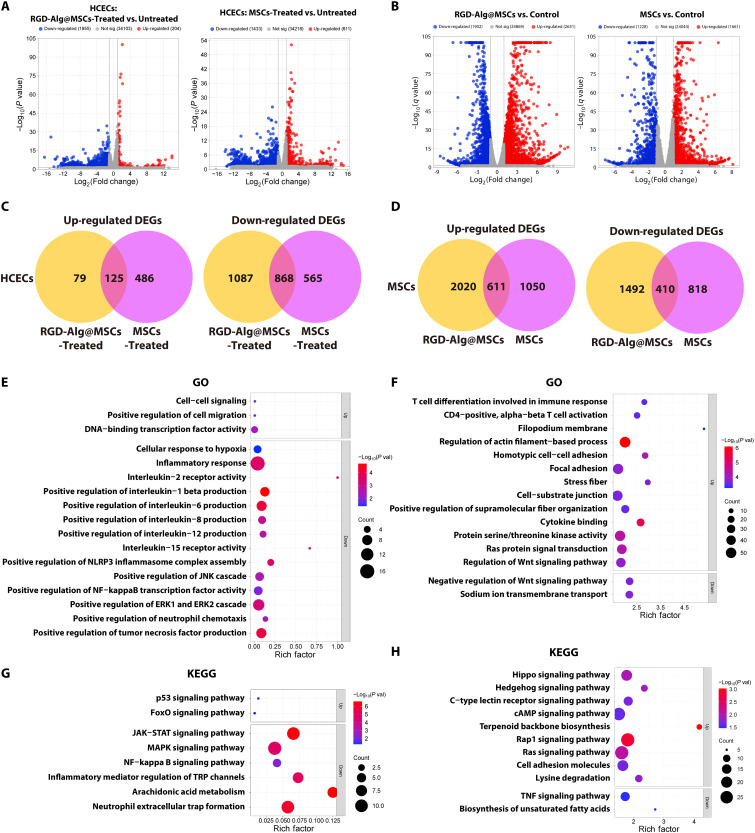
Transcriptomic analysis of HCECs and MSCs in coculture systems. (**A**) Volcano plots comparing gene expression in HCECs under hyperosmolarity across different treatment conditions, including RGD-Alg@MSCs-Treated versus Untreated and MSCs-Treated versus Untreated. Untreated refers to HCECs exposed to hyperosmolar conditions without any treatment. (**B**) Volcano plots comparing gene expression in MSCs across different groups, including RGD-Alg@MSCs versus Control and conventional MSCs versus Control. Control represents non-cocultured MSCs grown in 2D conditions. (**C**) Venn diagrams showing up-regulated/down-regulated DEGs in HCECs under hyperosmolarity after coculture with RGD-Alg@MSCs or MSCs, compared to the untreated group. (**D**) Venn diagrams showing up-regulated/down-regulated DEGs in MSCs of different groups compared to the control. (**E**) GO enrichment scatterplots and (**G**) KEGG pathway enrichment scatterplots of specific up-regulated and down-regulated DEGs identified in hyperosmotic-induced HCECs cocultured with RGD-Alg@MSCs. (**F**) GO enrichment scatterplots and (**H**) KEGG pathway enrichment scatterplots of specific up-regulated and down-regulated DEGs identified in RGD-Alg@MSCs. Bubble size represents gene count, and color indicates *P* value.

To understand the molecular mechanisms mediating therapeutic actions of MSCs encapsulated in microcarriers, we performed RNA-seq analysis on both MSCs and HCECs after coculture under hyperosmotic conditions. RNA was extracted from HCECs cocultured with either MSCs encapsulated in porous RGD-Alg microcarriers (RGD-Alg@MSCs-Treated) or MSCs (MSCs-Treated), as well as from the HCECs under hyperosmolarity without MSC coculture (Untreated). Simultaneously, RNA was extracted from MSCs in both RGD-Alg@MSC and MSC groups after coculture with HCECs under hyperosmolarity, with normal MSCs (not cocultured with HCECs) serving as the Control group. Differential expression analysis revealed distinct transcriptomic profiles between the treatment groups. In HCECs, the RGD-Alg@MSCs-Treated versus Untreated comparison identified 204 up-regulated and 1955 down-regulated genes, while the MSCs-Treated versus Untreated comparison yielded 611 up-regulated and 1433 down-regulated genes (Fig. 4A). Comprehensive functional enrichment analysis of the RGD-Alg@MSCs-Treated group and the MSCs-Treated group revealing respective molecular mechanisms is presented in fig. S17.

To investigate the unique molecular mechanisms associated with microcarrier-encapsulated MSCs, we performed Venn diagram analysis (*26*) of the up-regulated and down-regulated differentially expressed genes (DEGs) (Fig. 4C). This analysis revealed that the RGD-Alg@MSCs-Treated group exhibited 79 uniquely up-regulated and 1087 uniquely down-regulated DEGs that were not present in the MSCs-Treated group, suggesting specific molecular mechanisms that may contribute to the enhanced therapeutic efficacy of microcarrier-encapsulated MSCs.

To elucidate the unique biological significance of these DEGs, we performed Gene Ontology (GO) (Fig. 4E) and Kyoto Encyclopedia of Genes and Genomes (KEGG) pathway enrichment analyses (Fig. 4G), which revealed distinctive molecular signatures that highlight the enhanced therapeutic potential of RGD-Alg@MSCs over conventional MSCs. Up-regulated DEGs in RGD-Alg@MSCs were significantly enriched in biological processes related to cell-cell signaling and positive regulation of cell migration, alongside DNA binding transcription factor activity. These up-regulated functions represent a key advantage of the RGD-Alg microcarrier system, as they enhance the ability of MSCs to coordinate cellular responses during tissue repair and regeneration. The KEGG pathway analysis further identified unique up-regulation in p53 and FoxO signaling pathways, which confer superior cell survival, stress resistance, and cellular homeostasis compared to conventional MSCs. The most notable advantage of RGD-Alg@MSCs was demonstrated in their inflammatory profile. These microencapsulated MSCs exhibited substantial down-regulation of multiple inflammatory response pathways, including positive regulation of proinflammatory cytokines (IL-1β, IL-6, IL-8, IL-12), positive regulation of NLRP3 inflammasome complex assembly, and reduced neutrophil chemotaxis and TNF production. This represents a marked improvement over conventional MSCs in controlling excessive inflammation. Key inflammatory signaling pathways were also uniquely down-regulated, including Janus kinase (JAK)–signal transducer and activator of transcription (STAT), mitogen-activated protein kinase (MAPK), and nuclear factor κB (NF-κB) signaling pathways, collectively indicating superior immunomodulatory capabilities. Additionally, pathways involved in mediator regulation of transient receptor potential (TRP) channels, arachidonic acid metabolism, and neutrophil extracellular trap formation were more effectively suppressed in RGD-Alg@MSCs. Another distinctive advantage was the down-regulation of cellular response to hypoxia, suggesting that RGD-Alg@MSCs have improved adaptation to low-oxygen environments often present in damaged tissues, representing a critical advancement over conventional MSCs for therapeutic applications. These findings collectively indicate that RGD-Alg microcarrier system confers superior immunomodulatory properties to MSCs through the more effective suppression of multiple proinflammatory pathways and mediators while simultaneously enhancing cell migration and intercellular communication necessary for tissue repair. This enhanced immunoregulatory profile provides the molecular basis for understanding the superior therapeutic efficacy of RGD-Alg@MSCs over conventional MSCs in promoting tissue regeneration and dampening excessive inflammatory responses.

At the same time, we analyzed the transcriptomic changes in MSCs of different groups after coculture with HCECs. Compared to control MSCs, RGD-Alg@MSCs showed 2631 up-regulated and 1902 down-regulated genes, while MSCs exhibited 1661 up-regulated and 1228 down-regulated genes (Fig. 4B). The overall functional enrichment analysis of RGD-Alg@MSCs and MSCs is presented in fig. S18. Venn diagram analysis revealed that RGD-Alg@MSCs had 2020 uniquely up-regulated and 1492 uniquely down-regulated genes that were not shared with the MSC group (Fig. 4D).

The GO analysis (Fig. 4F) of uniquely up-regulated DEGs in RGD-Alg@MSCs revealed significant enrichment in T cell–related immune functions, including T cell differentiation involved in immune response and CD4-positive, α-β T cell activation. Additionally, up-regulation in cytokine binding further enhances the immunomodulatory capabilities of these cells through improved paracrine signaling. Structurally, RGD-Alg@MSCs exhibited up-regulation in cell adhesion and cytoskeletal organization processes including filopodium membrane, regulation of actin filament–based process, homotypic cell-cell adhesion, focal adhesion, stress fiber, cell-substrate junction, and positive regulation of supramolecular fiber organization. These structural adaptations reflect the advantages of three-dimensional (3D) microcarrier culture, which promotes enhanced cytoskeletal organization and cell-matrix interactions, likely contributing to improved cell survival and functionality. The KEGG pathway analysis (Fig. 4H) further demonstrated enrichment in multiple signaling cascades essential for cell communication and survival, including Rap1, Ras, and Hippo signaling pathways. Notably, the up-regulation of C-type lectin receptor signaling pathway is particularly important for immune regulation, as these receptors play crucial roles in immune cell recognition and response. The down-regulation of TNF signaling pathway in RGD-Alg@MSCs indicates enhanced anti-inflammatory properties, critical for reducing excessive inflammation in damaged tissues. The balance between up-regulated Wnt signaling pathway regulation and down-regulated negative regulation of Wnt signaling pathway suggests optimized control of this key developmental and regenerative pathway. These transcriptomic changes collectively explain the superior therapeutic efficacy of RGD-Alg@MSCs in tissue repair and immune modulation. The 3D microenvironment provided by the RGD-modified alginate microencapsulation appears to optimize the structural organization of MSCs, intercellular communication, and signaling networks, ultimately enhancing their immunomodulatory function and tissue regenerative potential when compared to conventional MSC cultures.

### Therapeutic effects of RGD-Alg@MSC eye drops in the DED mouse model

The in vivo biocompatibility was conducted before the therapeutic effect test. No toxicity was observed in the treated mice of all groups (fig. S19). To further evaluate whether our microcarriers might cause ocular discomfort or potential damage due to the size, TUNEL staining was performed on the eyeballs. No significant increase in TUNEL-positive cells was observed in the RGD-Alg@MSC–treated group compared to the control group, confirming the ocular safety of the RGD-Alg@MSC eye drops (fig. S20).

To evaluate ocular retention capabilities, we used red fluorescent protein–labeled MSCs (RFP-MSCs) for track. Conventional suspended RFP-MSCs or RGD-Alg@RFP-MSCs were topically administered to the ocular surface of mice and monitored using in vivo fluorescence imaging (fig. S21). Conventional RFP-MSCs showed rapid clearance from the corneal surface, with substantial signal reduction by 40 min and near-complete disappearance by 80 min post-application. In contrast, RGD-Alg@ RFP-MSCs demonstrated substantially enhanced retention at the ocular surface, with fluorescence signals maintained for up to 80 to 120 min after application. This substantially improved retention time suggests that RGD-Alg encapsulation provides a critical advantage by effectively prolonging the presence of therapeutic MSCs at the ocular surface, thereby extending their therapeutic window and enhancing treatment efficacy.

**Fig. 5.**
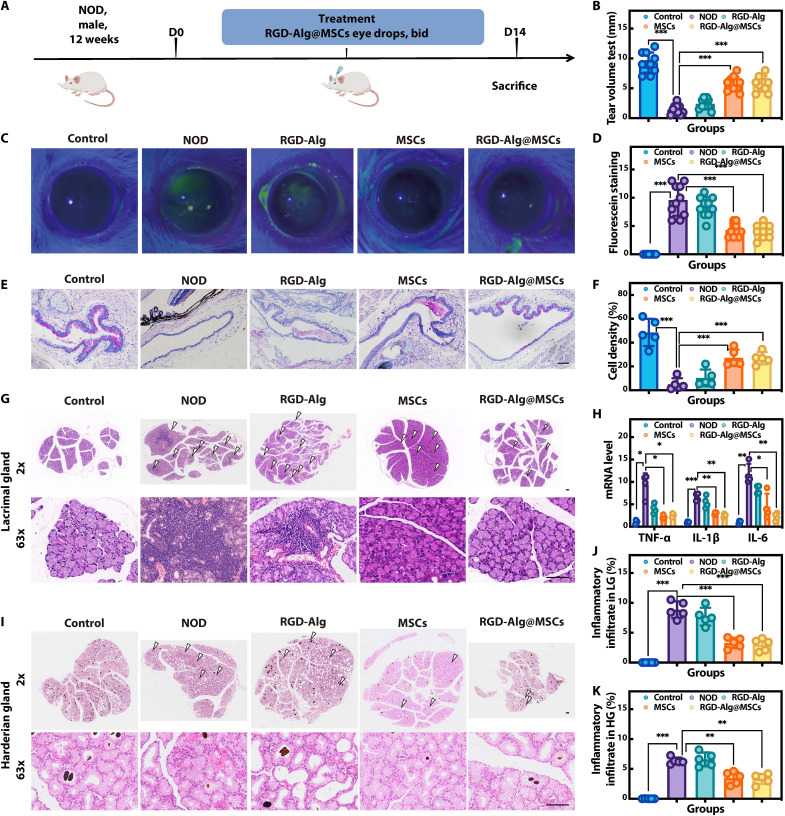
In NOD mice, the effectiveness of RGD-Alg@MSC eye drops was investigated. (**A**) Schematic description of animal experiment. (**B**) Tear volume measurements (*n* = 10) using cotton threads impregnated with phenol red. (**C**) Images of the fluorescein staining of the cornea. (**D**) Scores of the corneal fluorescein staining (*n* = 10). (**E**) Images of the conjunctival tissue stained by PAS. Scale bar, 50 μm. (**F**) Quantification of the conjunctival goblet cell density (*n* = 5) in the conjunctiva. (**H**) Typical inflammatory cytokine expression levels in the cornea and conjunctiva tissue (*n* = 4). H&E staining and histological analysis of the lacrimal glands (**G** and **J**) (*n* = 5) and harderian glands (**I** and **K**) (*n* = 5). Scale bar, 100 μm. White arrows point to the inflammatory infiltrate area.

To assess the efficacy of RGD-Alg@MSC eye drops on the autoimmune disease–related DED, we used 12-week male NOD mice (Fig. 5A). Male ICR mice of the same age with no treatment served as the control. As shown in our results, the untreated NOD mice exhibited significant symptoms of DED compared to the ICR control mice, including reduced tear production, increased corneal fluorescein staining, decreased conjunctival goblet cell density, and pronounced inflammatory infiltration in the lacrimal and harderian glands. These observations confirmed the presence of dry eye condition in our NOD mouse model, consistent with previous reports (*27*) of autoimmune-related DED. The administration of RGD-Alg@MSC eye drops successfully enhanced tear production (Fig. 5B) and decreased the score of sodium fluorescein staining (Fig. 5, C and D) in NOD mice. The treatment with RGD-Alg@MSCs increased the density of conjunctival goblet cells compared to no treatment (Fig. 5, E and F). We evaluated inflammatory cytokine mRNA expression in the ocular surface to learn more about whether RGD-Alg@MSCs can reduce inflammation in DED. The TNF-α, IL6, and IL-1β mRNA level of the cornea and conjunctiva tested by real-time quantitative polymerase chain reaction (RT-qPCR) analysis was shown to be lower in the RGD-Alg@MSC–treated mice as compared to the untreated group (Fig. 5H). Hematoxylin and eosin (H&E) staining was used on the lacrimal glands of NOD mice to assess the extent of inflammation infiltration (Fig. 5G), which is a crucial aspect of SS-like disease in the model used as well as a characteristic of autoimmune dacryoadenitis (*28*). Quantitative analysis of the H&E-stained sections (Fig. 5J) revealed significant differences in the degree of lymphocytic infiltration among the groups. The nontreatment and RGD-Alg groups exhibited extensive lymphocytic infiltration, characterized by densely packed lymphocyte clusters within the lacrimal gland tissue. Conversely, the MSC and RGD-Alg@MSC groups showed a marked reduction in lymphocytic infiltration, suggesting a substantial alleviation of autoimmune-induced inflammation. Likewise, RGD-Alg@MSC eye drops alleviated lymphatic infiltration in harderian glands in NOD mice (*29*) (Fig. 5, I and K), which were responsible for secreting tear lipids in mouse harderian glands. These findings confirmed the therapeutic efficacy of RGD-Alg@MSC eye drops in treating SS-like DED.

### Immunomodulatory effects of RGD-Alg@MSCs on DCs and T_H_17 differentiation in vitro

**Fig. 6.**
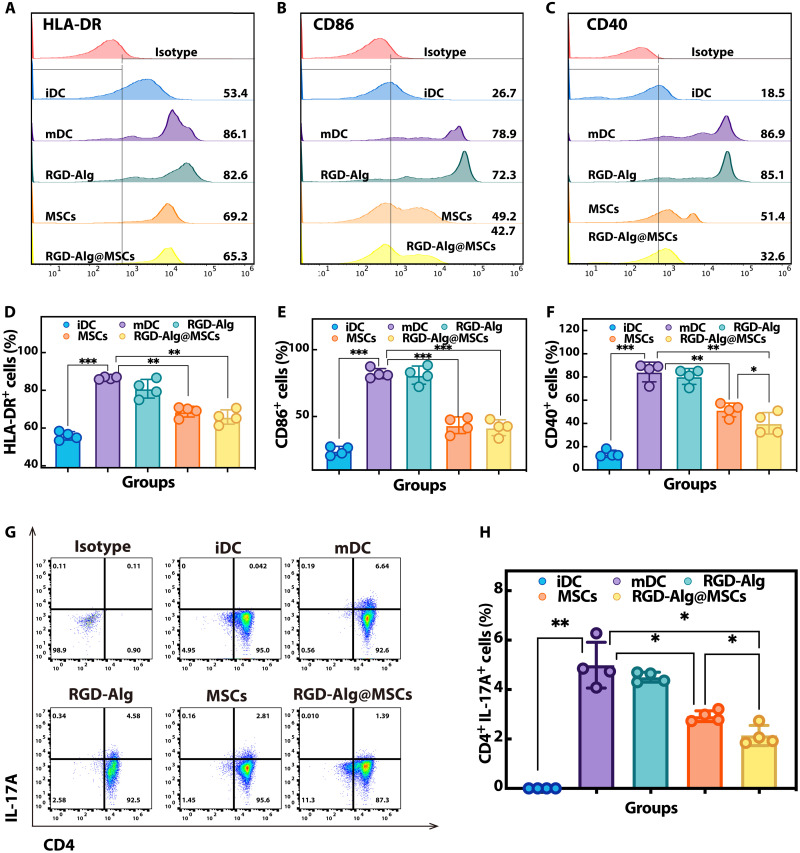
Effects of immune regulation resulted from RGD-Alg@MSCs in vitro. Flow cytometry showing the expression of representative cell surface markers HLA-DR (**A**), CD86 (**B**), and CD40 (**C**) of mDCs in different coculture treatment groups. The uninduced iDC group was used as the control. Quantification of the percentage of cells positively expressing these cell surface markers (**D** to **F**) (*n* = 4). (**G**) DCs treated with different methods were cocultured with naïve CD4^+^ T cells isolated from PBMCs, and flow cytometry was used to detect the proportion of T_H_17 cells (CD11c^−^ CD4^+^ IL-17A^+^) in them. (**H**) Percentage of T_H_17 cells (CD11c^−^ CD4^+^ IL-17A^+^ cells) (*n* = 4). Isotype, isotype control.

To explore the potential of RGD-Alg@MSCs in modulating immune responses, we designed in vitro experiments to assess their impact on DC maturation and T_H_17 differentiation based on reported studies (*26*, *30*). DCs were derived from human peripheral blood mononuclear cells (PBMCs). Immature DCs (iDCs) were generated by culturing PBMCs with interleukin-4 (IL-4) and granulocyte-macrophage colony-stimulating factor (GM-CSF), followed by stimulation with lipopolysaccharide (LPS) to induce their maturation into mature DCs (mDCs). These mDCs were then cocultured with RGD-Alg@MSCs using a Transwell chamber. To evaluate the expression of key surface markers, flow cytometry analysis was used. The successful induction of DCs was confirmed by low CD14 expression (fig. S22, A and D) and high CD11c expression (fig. S22, B and E) across all groups. Results showed that coculture with RGD-Alg@MSCs significantly reduced the expression of maturation markers HLA-DR (Fig. 6, A and D), CD86 (Fig. 6, B and E), and CD83 (fig. S22, C and F) compared to the untreated mDC group, indicating effective inhibition of DC maturation. Furthermore, RGD-Alg@MSCs decreased CD40 expression (Fig. 6, C and F), a critical molecule involved in DC–T cell interaction, significantly outperforming the simple MSC group. To assess the impact on T_H_17 differentiation, CD4^+^ naïve T cells were introduced after the MSCs were removed from the coculture. The RGD-Alg@MSC–treated DCs exhibited a significantly reduced ability to induce T_H_17 differentiation (Fig. 6, G and H), with a statistically lower proportion of CD4^+^ IL-17A^+^ cells compared to both the untreated mDCs and the MSC-only groups. These findings underscore the superior capacity of RGD-Alg@MSCs to inhibit DC maturation and suppress T_H_17 differentiation, disrupting the inflammatory loop associated with DED pathogenesis more effectively than conventional MSCs alone.

### Immunomodulatory effects of RGD-Alg@MSCs in vivo

**Fig. 7.**
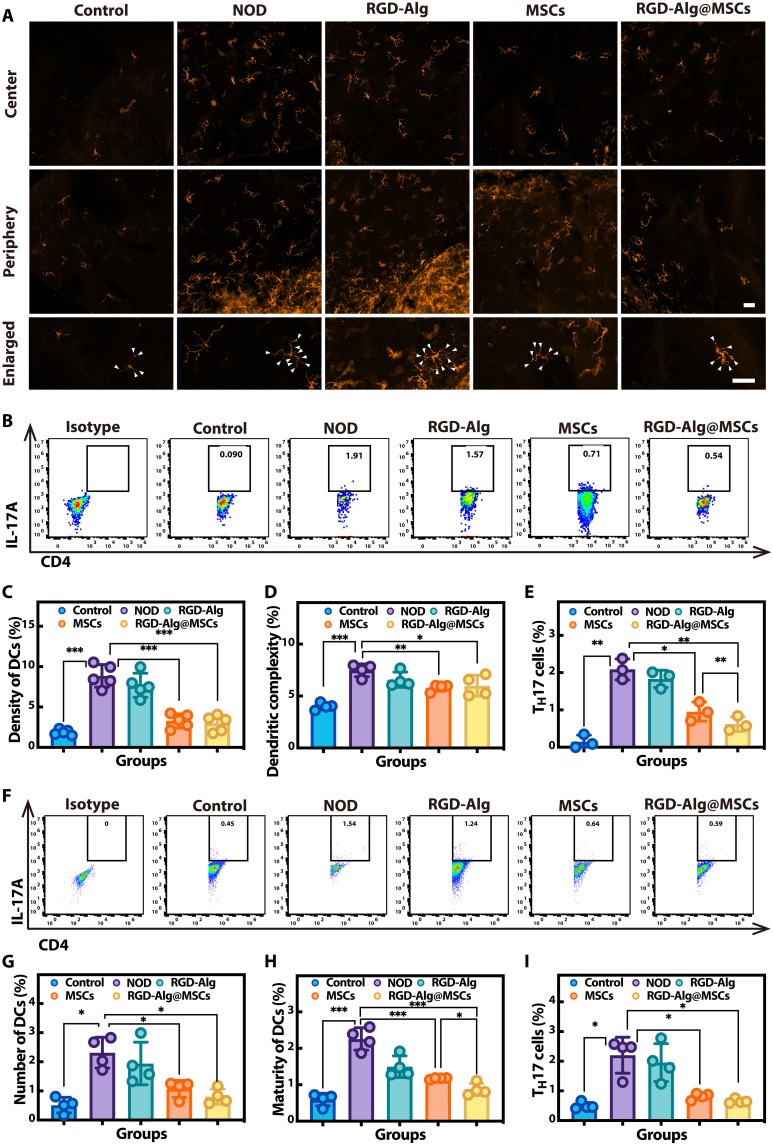
Effects of immune regulation resulted from RGD-Alg@MSC eye drops in NOD mice. (**A**) Representative images of corneal DCs in different groups (scale bar, 50 μm). (**B**) Flow cytometry showing the level of T_H_17 cells in the cornea. Quantification of DC density (**C**) (*n* = 5) and dendritic complexity (**D**) (*n* = 4) in the cornea. (**E**) Quantification of T_H_17 cell percentage in the cornea (*n* = 3) via flow cytometry. (**F**) Flow cytometry showing the level of T_H_17 cells in the dLNs. Quantification of cell number (**G**) and maturity (**H**) of DCs in dLNs (*n* = 4). (**I**) Percentage of T_H_17 cells in dLNs (*n* = 4). The number of dendritic points per cell serves as a proxy for the dendritic complexity of DCs.

Next, we evaluated the effects of RGD-Alg@MSCs on the immune responses in the cornea and ocular draining lymph nodes (dLNs) in NOD mice. Following topical treatment with RGD-Alg@MSCs, RGD-Alg, MSCs, or no treatment at all, the corneas and dLNs were harvested. To determine how many DCs were in each cornea, we performed immunofluorescence staining. Using flow cytometry, the number and the maturity of DCs in dLNs were evaluated. The results of immunofluorescence staining (Fig. 7A) demonstrated a significant rise in dendritic maturity and DC density in the cornea of NOD mice. Treatment with RGD-Alg@MSC eye drops significantly reduced the density and dendritic maturity of DCs compared to the NOD mice with no treatment group (Fig. 7, C and D). Flow cytometric analysis further elucidated the immunomodulatory impact of RGD-Alg@MSCs. In the cornea of NOD mice, the percentage of T_H_17 cells was remarkably high, which are essential to the pathophysiology of DED (*31*) (Fig. 7, B and E). However, treatment with RGD-Alg@MSCs substantially decreased the percentage of T_H_17 cells in the cornea, significantly outperforming the reductions observed with MSCs alone. This finding suggested that the RGD-Alg@MSC eye drops more effectively mitigate the inflammatory response associated with T_H_17 cells in DED. In addition, we checked the immunomodulatory impact in the dLNs. As in many previous studies (*32*, *33*), we used the percentage of CD45^+^CD11c^+^MHC-II^+^ cells in lymph nodes to represent the quantity of DCs, and the percentage of CD45^+^CD11c^+^CD86^+^ cells to represent the maturity of DCs. Treatment with RGD-Alg@MSCs significantly down-regulated both the number (Fig. 7G and fig. S23A) and maturity (Fig. 7H and fig. S23B) of DCs in the dLNs, with a particularly pronounced effect on DC maturation compared to conventional MSC treatment. In the dLNs of NOD mice, there was a considerable rise in the percentage of CD4^+^IL-17A^+^ T_H_17 cells (Fig. 7, F and I). This percentage was significantly reduced by RGD-Alg@MSC treatment, demonstrating the strong immunoregulatory effect of the treatment modality. Overall, these results demonstrate that RGD-Alg@MSC eye drops exert a substantial immunomodulatory effect in NOD mice, more potently than conventional MSCs in reducing both DC maturation and T_H_17-mediated inflammation, which are key contributors to the disease pathology (*34*, *35*).

## DISCUSSION

Here, we developed an MSC-encapsulated porous microcarrier eye drop system for the treatment of autoimmune DED. Our key findings demonstrate that RGD-Alg porous microcarriers encapsulating MSCs exhibited significant therapeutic effects both in vitro and in vivo. Under hyperosmotic conditions in vitro, RGD-Alg@MSCs enhanced corneal epithelial cell viability, reduced apoptosis and ROS levels, and decreased proinflammatory cytokine expression. In the NOD mouse model of autoimmune-related DED, RGD-Alg@MSCs significantly increased tear production, promoted corneal healing, restored conjunctival goblet cells, decreased proinflammatory cytokine expression in the cornea and conjunctiva, and alleviated inflammatory infiltration in the lacrimal and harderian glands.

Our study demonstrated the advantages of microcarrier technology for stem cell therapy in DED. Compared to traditional MSC delivery methods, the RGD-Alg@MSC system addresses several critical challenges: (i) The porous structure and RGD modification enhance MSC adhesion, survival, and function while providing a protective microenvironment for sustained therapeutic factor release; (ii) RGD-Alg@MSCs demonstrate significantly stronger immunomodulatory capabilities than conventional MSCs, more effectively reducing DC maturation and T_H_17 cell infiltration both in vitro and in vivo; (iii) transcriptomic analysis revealed distinct gene expression patterns in RGD-Alg@MSC–treated HCECs, with unique enrichment in cytokine-receptor interaction and IL-17 signaling pathways; (iv) the system achieves prolonged retention in a noninvasive eye drop form; and (v) the microcarrier platform enables efficient large-scale expansion of MSCs while preserving their stemness. These findings not only provide a therapeutic strategy for autoimmune-related DED but also establish a platform for cell expansion and delivery for cell therapy in ocular surface diseases.

Our findings reveal several key mechanisms by which RGD-Alg@MSCs alleviate DED symptoms. The porous microcarrier structure provides a protected microenvironment for MSCs while allowing efficient exchange of nutrients and therapeutic factors with the surrounding tissue. This is evidenced by the sustained release of therapeutic factors including TSG-6, CCL-20, IDO, and PGE_2_ observed in our in vitro studies, which are crucial for immunomodulation, anti-inflammatory effects and tissue repair. Our immunological analyses demonstrated that RGD-Alg@MSCs more effectively modulate the immune environment in DED compared to conventional MSCs. In vitro coculture experiments showed that the RGD-Alg@MSC group exhibited a stronger reduction in CD40 expression on mDC surfaces and more potently inhibited mDC-induced differentiation of CD4^+^ naïve T cells into T_H_17 cells. These enhanced effects were confirmed in vivo, where RGD-Alg@MSC treatment was significantly more effective at reducing both the number and maturity of DCs in the dLNs and decreasing corneal T_H_17 cell infiltration compared to conventional MSCs. This represents a notable advantage as the microcarrier system enhances the immunomodulatory capacity of MSCs, leading to more effective disruption of the inflammatory feedback loop characteristic of DED.

Furthermore, transcriptomic analysis revealed distinct gene expression patterns in HCECs cocultured with RGD-Alg@MSCs compared to those cocultured with conventional MSCs. The RGD-Alg@MSCs-Treated group exhibited unique molecular signatures not present in the MSCs-Treated group, particularly in immunomodulatory pathways and inflammatory responses. Specifically, GO and KEGG pathway analyses demonstrated enhanced down-regulation of proinflammatory cytokines (IL-1β, IL-6, IL-8, IL-12) and signaling pathways (JAK-STAT, MAPK, and NF-κB) in the RGD-Alg@MSC group. Simultaneously, pathways related to cell migration, intercellular communication, and stress resistance were uniquely up-regulated. The MSCs themselves showed significant transcriptomic differences when cultured in the porous RGD-Alg microcarriers, with up-regulation in T cell immunomodulation functions, cytokine binding, and cell-matrix interactions, while showing down-regulation of TNF signaling pathways. These molecular changes collectively provide the mechanistic basis for the enhanced therapeutic efficacy of RGD-Alg@MSCs, highlighting how the 3D microenvironment optimizes MSC function for both immunomodulation and tissue regeneration.

The careful formulation of our MSC-encapsulated porous microcarrier eye drops was optimized specifically for DED treatment. We selected alginate as the primary material due to its biocompatibility, minimal immunogenicity, and mucoadhesive properties that extend ocular surface residence time. RGD peptide modification enhanced cell attachment and survival through integrin receptor interactions. CaCl_2_ was chosen as the crosslinking agent for its rapid ionic bonding with alginate, creating a gentle gelation process that preserves MSC viability while providing mechanical stability without introducing toxic chemical crosslinkers. PEO, which shares chemical structure with U.S. Food and Drug Administration–approved polyethylene glycol (PEG) but with higher molecular weight, served as a temporary water-soluble porogen mixed in the precursor solution. After gelation, it was removed by dissolution when transferred to water, creating essential porous structures while avoiding residual toxicity and harsh processing conditions typically associated with conventional porogens. The microcarrier was suspended in isotonic PBS to ensure stability and minimize irritation.

The physical properties of our microcarriers contribute substantially to their ocular applicability. With a diameter of 130 μm, they fall within the standard size range for cell microcarriers (100 to 300 μm) used in biomedical applications. Their measured Young’s modulus of approximately 50 kPa indicates a soft, elastic nature comparable to soft biological tissues, potentially minimizing foreign body sensation while maintaining structural integrity. The rheological properties further support their suitability for ocular use. Despite instrument limitations preventing assessment at the full range of physiological blinking shear rates (which can reach up to 100,000 s^−1^), our measured shear-thinning behavior suggests that viscosity would likely decrease further at higher shear rates during actual blinking. With viscosities within the optimal 30 to 300 mPa·s range for ophthalmic formulations (*17*), our microcarriers achieve a rheological balance that conventional eye drops between retention time and patient comfort.

For the eye drop formulation, we prepared a suspension of microcarriers in PBS at a concentration of 6 × 10^7^ MSC-laden microcarriers per milliliter, administering 5 μl per eye twice daily. In our protocol, freshly prepared microcarriers were used immediately for maximum therapeutic efficacy, with storage at 4°C maintained throughout the transfer process. Additionally, we conducted stability studies, which demonstrated that RGD-Alg@MSCs maintained structural integrity and proper cell viability for at least 24 hours when stored at 4°C, showing superior stability compared to MSCs in direct suspension, suggesting potential for practical clinical application with storage advantages. TUNEL staining confirmed the ocular safety of RGD-Alg@MSC eye drops, as no significant apoptosis was observed in the eyeballs of treated healthy ICR mice, further supporting their biocompatibility for ocular applications.

Compared to existing DED therapies, our approach offers several advantages. Unlike artificial tears that provide only temporary symptomatic relief, RGD-Alg@MSCs address the underlying inflammatory mechanisms driving DED pathogenesis. Conventional MSC therapies face challenges in cell survival and retention on the ocular surface, whereas our microcarrier system demonstrated improved MSC viability and prolonged therapeutic effects. MSC-conditioned medium faces substantial limitations including the presence of nonspecific metabolites and waste products, lacks the dynamic responsiveness to the disease microenvironment, and encounters standardization challenges. In contrast, our approach overcomes these limitations through sustained delivery of viable cells that can actively respond to the pathological conditions. The noninvasive nature of our eye drop formulation also represents an advantage over surgical interventions or direct intraocular injections, potentially improving patient compliance and reducing procedural risks. Our system enhances the therapeutic potential of MSCs by optimizing their microenvironment, particularly suitable for the long-term management of chronic inflammatory conditions like DED.

Despite the promising results, our study has several methodological limitations that must be acknowledged. First, while we demonstrated the efficacy of RGD-Alg@MSCs in alleviating DED symptoms in the NOD mouse model, future studies should evaluate long-term effects and durability of the therapeutic response. The NOD mouse model, while valuable for studying autoimmune-related DED due to its spontaneous development of lacrimal gland inflammation and ocular surface damage, may not fully recapitulate all aspects of human DED, which is often multifactorial. Alternative models, such as the scopolamine-induced DED model that simulates non-Sjögren’s aqueous-deficient dry eye, or the age-related dry eye, could be explored in future studies to evaluate the therapeutic potential of our approach across diverse DED etiologies.

In our mechanism exploration, our in vitro hyperosmotic HCEC model cannot fully replicate the complexity of DED or incorporate other components of the lacrimal functional unit such as lacrimal glands. Additionally, while RNA-seq effectively distinguished molecular responses in both MSCs and HCECs, the absence of proteomic analysis limits direct characterization of the MSC secretome. However, this transcriptomic approach provided valuable insights by clearly delineating the distinct responses in both cell populations. In comparing RGD-Alg@MSCs with conventional MSCs, we focused on uniquely regulated genes rather than analyzing more complex expression patterns (such as genes up-regulated in one group but down-regulated in another). This analytical approach was intentionally designed to prioritize the most statistically significant and consistent pathways, maintain analytical clarity, and avoid potential confounding effects from more variable expression patterns.

Additionally, further investigation into the optimal dosing regimen, concentration, and frequency, long-term storage stability, and scalable manufacturing processes of RGD-Alg@MSCs will be essential for successful clinical translation. Future formulation refinements could explore alternative vehicles such as artificial tear solutions or hyaluronic acid to potentially enhance retention time and therapeutic efficacy while maintaining the fundamental microcarrier design. Our transcriptomic analysis has identified several potential molecular pathways involved in the therapeutic effects of RGD-Alg@MSCs, which warrant further investigation to develop more targeted approaches for DED treatment.

## MATERIALS AND METHODS

### Materials

The chemicals used in this study included sodium alginate (Sigma-Aldrich, USA), RGD peptide (Typeptide Biotechnology, China), PEO (Sigma-Aldrich), CaCl_2_ (Sigma-Aldrich), EDC (Aladdin, China), NHS (Aladdin), and sodium citrate (Sigma-Aldrich). The water used in the experiment underwent purification through the Millipore Milli-Q purification system.

### Cells

HCECs (CRL-11135), human umbilical cord MSCs (Nanjing Drum Tower Hospital), and RFP-MSCs (Nanjing Drum Tower Hospital) were both cultured in the Dulbecco’s modified Eagle’s medium/F12 medium (HyClone, Cytiva) containing 10% fetal bovine serum (FBS; Gibco) and 1% penicillin/streptomycin (PS; Pricella). The extraction of PBMCs (*36*) and the induction processes for iDCs and mDCs (*37*) followed a similar protocol to the previous article. Briefly, IL-4 (PeproTech) and GM-CSF (PeproTech) were used to induce iDCs from PBMCs, while LPS (Sigma-Aldrich) was added to generate mDCs. Naïve CD4^+^ T cells were isolated from PBMCs using magnetic bead sorting (ImunoSep) and cultured in the medium containing plate-bound anti-CD3 (Thermo Fisher Scientific), soluble anti-CD28 (Thermo Fisher Scientific), anti-IL-4 (Abcam), and anti-IFN-γ (Interferon gamma; Abcam) antibodies, along with IL-23 (interleukin-23; PeproTech), TGF-β (transforming growth factor-β; PeproTech) and IL-6 (PeproTech) for T_H_17 differentiation followed a similar protocol before (*36*, *38*). In RPMI 1640 (HyClone), PBMCs, iDCs, mDCs, and naïve CD4^+^ T cells were cultivated with 10% FBS and 1% PS added. All cells were incubated at 37°C with 5% CO_2_ in a humidified environment.

### Synthesis and analysis of RGD-Alg hydrogel

RGD-Alg hydrogel was fabricated by adapting established protocols to meet specific experimental needs (*15*). Sodium alginate, at a concentration of 0.12 g in 10 ml of PBS buffer (pH 6 to 6.5, Servicebio), was activated using an equimolar mixture of EDC and NHS. RGD peptide was then introduced, and the mixture was stirred continuously to promote coupling. Following the reaction, the solution was dialyzed using a 14-kDa cutoff membrane (Biosharp) and then neutralized to achieve a pH of 7 to 7.5. Subsequent lyophilization yielded a dry powder, which was then rehydrated with deionized water to obtain a specific concentration of the RGD-Alg precursor solution as required. ^1^H NMR spectroscopy (Varian Inova 500 MHz) was used to identify the characteristic peaks corresponding to the RGD moiety integrated within the alginate structure. Using an FTIR system (Bruker, USA), the integration of RGD peptides into the alginate hydrogel was evaluated via FTIR over a 400 to 4000 cm^−1^ spectrum at a 2 cm^−1^ resolution.

### Cell adhesion in RGD-modified hydrogel

To assess cell adhesion on RGD-modified alginate hydrogels, 2% RGD-Alg solution was cured in 2% CaCl_2_ to form hydrogels. After lyophilization, the films were seeded with MSCs (*39*). Cells were stained with calcein AM (C2015, Beyotime) and examined after a 24-hour period.

### Fabrication of the porous RGD-Alg hydrogel

A 2% RGD-Alg solution (initial concentration selected to control variables while exploring PEO effects) with a final concentration of 0.01%, 0.05%, 0.1%, 0.5%, 1%, 1.5%, and 2% PEO was crosslinked in a 2% CaCl_2_ solution. This CaCl_2_ concentration was selected based on established protocols (*40*, *41*). Previous studies confirmed that RGD modification did not significantly alter these optimal crosslinking conditions (*42*, *43*, *44*). After freeze-drying, the surface pore size was observed using SEM.

### Swelling characterization of the porous RGD-Alg microcarriers

The swelling properties were characterized by the swelling of the porous RGD-Alg microcarriers. Wd, the appropriate dry weight, was first obtained by lyophilizing the microcarriers. Each sample was immersed in deionized water, and the sample weight was recorded daily until the sample weight became equal to the swelling equilibrium weight, Weq. This procedure was repeated three times under the same conditions. Swelling ratio (%) = (Weq/Wd) × 100% was the formula used to estimate the swelling ratio (*45*).

### Optimization of electrospraying parameters

The pre-gel solution was electrosprayed into a 2% CaCl_2_ solution under varying parameters: voltage (4 to 12 kV), flow rate (50 to 400 μl/min), and RGD-Alg concentration (1.5% to 4%). Their appearance was observed under a light microscope, and ImageJ software was used to measure and analyze particle size distribution under different parameters.

### Mechanical testing of microspheres

The mechanical properties of the microspheres were evaluated using a micrometer-scale mechanical testing system (Microsquisher, CellScale) (*46*). A tungsten cantilever beam (modulus = 411 GPa, diameter = 203.2 μm) was used to compress the microspheres. All samples were tested in PBS at 37°C. Young’s modulus was determined at 25% deformation following a previously published method (*47*). The force at 25% deformation was calculated via linear regression of the force-displacement curve.

### Rheological characterization test

The rheological properties of 1.5% PVA (Aladdin), 0.3% sodium hyaluronate (Aladdin), 0.5% CMC solutions (Aladdin), and RGD-Alg@MSCs were characterized using the Rotational Rheometer (Thermo Fisher Scientific HAAKE MARS iQ) equipped with a parallel plate geometry (*48*). All measurements were conducted at 37°C in a hydrated environment to mimic physiological conditions. Steady shear tests were conducted over a shear rate range of 350 to 2350 s^−1^ to evaluate the viscosity (η) and shear-thinning behavior of the solutions. Each solution was tested in triplicate, and data were analyzed using HAAKE RheoWin software.

### Biocompatibility test

To assess biocompatibility, MSCs and HCECs were cultured with extracts from the porous microcarriers for 48 hours. Calcein AM and PI (C2015, Beyotime) were used to stain the cells, which were then photographed. The fluorescence was measured, and the cell viability and proliferation were assessed (*49*). The proportion of live cells was determined through fluorescence quantification.

### Fabrication of MSC-encapsulated porous RGD-Alg hydrogel microspheres

A 2% RGD-Alg solution containing 0.1% PEO and 6 × 10^7^ MSCs/ml was prepared. This cell-laden solution was processed using an electrospray device with a high-voltage power supply and a syringe pump operating at a voltage of 8 kV and a flow rate of 100 μl/min (*50*, *51*). The solution was electrosprayed and crosslinked in a 2% CaCl_2_ solution to form microcarriers. After 5 min, microcarriers were harvested and then soaked in PBS to remove PEO, resulting in porous RGD-Alg microcarriers encapsulated with MSCs (RGD-Alg@MSCs).

### Assessment of MSC viability and proliferation in the porous RGD-Alg microcarriers

RGD-Alg@MSCs were cultured for 1, 3, and 7 days. Post-incubation, the RGD-Alg@MSCs were examined under fluorescence microscopy. Porous Alg microcarriers with MSCs served as the control. Calcein AM and PI were used to stain the cells. By evaluating the intensity of cell fluorescence, the viability and the proliferation rate of the MSCs were objectively calculated (*39*).

### Assessment of MSC stemness in the porous RGD-Alg microcarriers

RGD-Alg@MSCs were dissolved using a mild, nonirritating 55 mM sodium citrate solution, followed by centrifugation at 800 rpm for 5 min to collect the encapsulated MSCs without compromising cell viability or functional state (*52*, *53*). Using flow cytometry, cell surface marker expression was examined to assess the stemness preservation of MSCs grown inside the hydrogel microcarriers. Following 1 week of culture, MSCs were extracted from the microcarriers and stained with antibodies that were fluorescently tagged against CD105 (BioLegend), CD90 (BioLegend), CD73 (BioLegend), CD45 (BioLegend), CD34 (BioLegend), CD11b (BioLegend), and CD19 (BioLegend) (*54*). Flow cytometry was performed using the FACSCalibur system (BD, USA). FlowJo (version 10.4) was used for data analysis.

### Assessment of RGD-Alg@MSC eye drop stability

The RGD-Alg@MSC microcarriers were suspended in PBS to prepare eye drops and stored at 4°C for immediate use. To evaluate the storage stability, we assessed the morphological integrity of the eye drops during a 24-hour period. Samples were collected at 0, 12, and 24 hours and observed under a light microscope (Nikon, Japan) to assess the preservation of the spherical morphology over time (*55*, *56*).

The viability of MSCs within the stored microcarriers was assessed after 24 hours of storage at 4°C in PBS. RGD-Alg@MSCs were dissolved using 55 mM sodium citrate solution, followed by centrifugation to collect the encapsulated MSCs. Cell viability was evaluated using flow cytometry with calcein AM/PI staining. Three conditions were compared: (i) MSCs cultured under standard conditions (37°C, complete medium), (ii) MSCs in PBS at 4°C, and (iii) RGD-Alg@MSCs stored in PBS at 4°C.

### In vitro DED model

To mimic an in vitro DED model in HCECs, a medium (450 mOsm/liter) was prepared by supplementing NaCl (Shanghai Hushi), following the previous method (*57*).

### Evaluation of RGD-Alg@MSCs for DED treatment in vitro

To simulate DED in vitro, the cells were subjected to hyperosmotic stress in a modified medium. Then, RGD-Alg@MSCs were inserted into the upper inserts of the Transwell system (Labselect) to coculture with the HCECs for 24 hours (*58*). Following the cocultivation procedure, a set of experiments was carried out to examine the potential therapeutic benefits of RGD-Alg@MSCs.

### Cell viability recovery test

The vitality of HCECs was tested with an assay of cell counting kit-8 (CCK-8) (Vazyme) after a 24-hour coculture (*20*). The optical density at 450 nm was measured using a microplate reader (Thermo Fisher Scientific), in accordance with protocol from the manufacturer for the test.

### Evaluation of apoptosis rate

Using an apoptosis kit (Abbkine), an in vitro evaluation was carried out to determine the capacity of RGD-Alg@MSCs to reduce apoptosis (*59*). Flow cytometry using the BD Accuri C6 (BD Biosciences, USA) was used. The analysis was done by FlowJo. Figure S24 depicts the flow cytometry gating rule.

### Real-time quantitative PCR

TRIzol reagent (Invitrogen, USA) was used to extract the total RNA from cells as well as corneal and conjunctival tissues (*57*). One microgram of RNA was reverse-transcribed into complementary DNA (cDNA) using reverse transcription kit (Vazyme). The resulting cDNA was then amplified through RT-qPCR on the Q5 system using a SYBR Green-based master mix (Vazyme). A set of primers was displayed in table S1.

### Assessment of cytokine release

To quantify the release of cytokines specifically from MSCs in the porous RGD-Alg microcarriers, both single-culture and coculture experiments were conducted. MSCs alone or RGD-Alg@MSCs alone were cultured in complete medium as controls, while parallel coculture experiments with HCECs were performed as described previously. Supernatants from both conditions (single culture and coculture) were collected after a 24-hour period and analyzed for the presence of therapeutic factors including TSG-6 (*60*, *61*, *62*), CCL20 (*63*), IDO (*64*, *65*), and PGE_2_ (*66*), and an enzyme-linked immunosorbent test (ELISA) was used. Supernatants were collected and subjected to specific ELISA kit analysis following a 24-hour coculture period (table S2).

### RNA-seq and bioinformatics analysis

HCECs and MSCs were cocultured in a Transwell system under hyperosmotic conditions to investigate the therapeutic effects of RGD-Alg@MSCs on HCECs and explore the potential molecular mechanisms mediating these actions. For corneal epithelial cells, the treatment groups included cells cocultured with RGD-Alg@MSCs (RGD-Alg@MSCs-Treated) or 2D-cultured MSCs (MSCs-Treated), while the disease control group consisted of HCECs under hyperosmotic conditions without MSC coculture (Untreated). For MSCs, the treatment groups included RGD-Alg@MSCs (RGD-Alg@MSCs) and 2D-cultured MSCs (MSCs) cocultured with HCECs under hyperosmotic conditions, with normal MSCs (not cocultured with HCECs under hyperosmotic conditions) serving as the baseline control (Control). HCECs from the RGD-Alg@MSCs-Treated, MSCs-Treated, and Untreated groups, as well as MSCs from the RGD-Alg@MSCs, MSCs, and control MSC groups, were collected for RNA extraction.

Total RNA was extracted using TRIzol reagent (Invitrogen) following the protocol of the manufacturer. RNA quantity and purity were assessed with NanoDrop ND-1000 (NanoDrop, USA), and integrity was confirmed using Bioanalyzer 2100 [Agilent, USA; RNA integrity number (RIN) > 7.0] and denaturing agarose gel electrophoresis. Poly(A) mRNA was isolated from 1 μg of total RNA using Dynabeads Oligo (dT)25-61005 (Thermo Fisher Scientific, USA) with two purification rounds, fragmented at 94°C for 5 to 7 min using the Magnesium RNA Fragmentation Module [New England Biolabs (NEB); catalog no. E6150, USA], and reverse-transcribed into cDNA with SuperScript II Reverse Transcriptase (Invitrogen, catalog no. 1896649, USA). Second-strand DNA was synthesized using *Escherichia coli* DNA polymerase I (NEB, catalog no. M0209, USA), ribonuclease H (NEB, catalog no. M0297, USA), and dUTP solution (Thermo Fisher Scientific, catalog no. R0133, USA). After adding A-bases to the blunt ends, indexed adapters were ligated, and size selection was performed using AMPureXP beads. The U-labeled second-strand DNA was treated with UDG enzyme (NEB, catalog no. M0280, USA), followed by PCR amplification (initial denaturation at 95°C for 3 min; eight cycles of 98°C for 15 s, 60°C for 15 s, 72°C for 30 s; final extension at 72°C for 5 min). The resulting cDNA libraries [average insert size: 300 ± 50 base pairs (bp)] were sequenced on an Illumina NovaSeq 6000 (LC-Bio Technology Co. Ltd., Hangzhou, China) using 2 × 150 bp paired-end sequencing (PE150) (*67*).

Raw sequencing data were quality-filtered using fastp (default parameters) to remove adapters, low-quality reads, and undetermined bases. Clean reads were aligned to the *Homo sapiens* GRCh38 reference genome using HISAT2 (*68*). Transcripts were assembled and merged across samples using StringTie (*69*) and gffcompare, respectively. Gene expression levels were quantified as FPKM (fragments per kilobase of transcript per million mapped reads) using StringTie. Differential expression analysis was performed with the R package edgeR (*70*), defining DEGs as those with a fold change of >2 or <0.5 and *P*-adjust value of <0.05 using the Benjamini-Hochberg correction method. The DEGs were subjected to GO (*71*) and KEGG (*72*) pathway enrichment analysis using DAVID software, with a significance threshold of *P* < 0.05.

### Coculture of RGD-Alg@MSCs and DCs

After inducing the maturation of DCs, a Transwell chamber was placed into the well plate. The upper layer of the chamber was loaded with equal amounts of RGD-Alg empty vector, conventionally cultured MSCs, and RGD-Alg@MSCs. The coculture was conducted for 24 hours, with the uninduced iDC group serving as the control. To evaluate the expression of DC surface markers including CD14, CD11c, CD83, CD86, human leukocyte antigen (HLA)–DR, and CD40, flow cytometry was used (*30*).

### Coculture of treated DCs with naïve CD4^+^ T cells

After the DC coculture was finished, treated DCs were collected, washed with PBS, and cocultured at a 1:10 ratio with naïve CD4^+^ T cells (*30*, *73*). To encourage the development of T_H_17 cells, the T cells were stimulated with plate-bound anti-CD3 and soluble anti-CD28 antibodies, and the medium was supplemented with a cytokine cocktail of IL-23, TGF-β and IL-6, along with neutralizing antibodies against |L-4 and IFN-γ. The coculture lasted for 72 hours. The percentage of T cells that developed into T_H_17 cells in each treatment group was assessed using flow cytometry, following the gating strategy for T_H_17 cells (CD11c^−^ CD4^+^ IL-17A^+^), as illustrated in fig. S25.

### DED mouse model and treatment

To assess the efficacy of RGD-Alg@MSC eye drops, we used 12-week-old male NOD mice (Huachuang Sino), which naturally develop symptoms consistent with autoimmune DED. Male NOD mice were specifically selected because they exhibit more severe lacrimal gland dysfunction compared to females (*74*), with pronounced CD4^+^ T cell infiltration leading to acinar atrophy and ductal obstruction. This model is well established for studying SS-like DED, characterized by reduced tear secretion, corneal epithelial damage, conjunctival goblet cell loss, and inflammatory infiltration of the lacrimal glands. The pathological changes in this model closely resemble the dysfunction of the lacrimal functional unit observed in human DED patients, making it suitable for evaluating therapeutic interventions (*27*).

The mice were assigned to four groups of 15 by blinded randomization. Eye drops containing 5 μl of either RGD-Alg@MSCs (containing about 3 × 10^5^ MSCs), porous RGD-Alg microcarriers, or suspended MSCs (containing about 3 × 10^5^ MSCs) were applied twice daily for 14 days in three groups under different treatments. The left group that received no treatment was NOD group. Furthermore, a group of male ICR mice of the same age (Huachuang Sino) kept in the same setting with no modifications served as the control for NOD mice. The study followed the guidelines established by the ethics committee of Nanjing Drum Tower Hospital (permission number 2022AE01039).

The effectiveness of RGD-Alg@MSC eye drops was assessed using fluorescein staining, where standard sodium fluorescein strips (Tianjin Jingming) moistened with 200 μl of sterile water were gently applied to the mouse corneal surface and evaluated 2 min post-application, yielding a score between 0 and 15 (*75*). Tear volume was measured using phenol red thread test, which was selected for its established advantages in small animal models including minimally invasive application, precise measurement capability, and suitability for the small palpebral fissure size of mice compared to standard Schirmer strips (*76*). Eye photography was also used to document changes. After 14 days of treatment, ocular tissues were collected for periodic acid–Schiff (PAS) staining and H&E staining to examine the structural alterations in corneal tissue.

### PAS staining and H&E staining

The specimens that were gathered were preserved in a solution of 4% paraformaldehyde (PFA; Servicebio). After that, these specimens were immersed in paraffin and then sectioned into slices. PAS staining was performed on the slices of the eyeballs and eyelids using a kit from Solarbio. The sections of lacrimal glands and harderian glands underwent H&E staining using a kit from Servicebio. Nikon digital light microscope was used to take and evaluate images of the stained sections (*57*).

### In vitro biocompatibility studies

To evaluate the in vivo biocompatibility of RGD-Alg@MSC eye drops, healthy male ICR mice (12 weeks old, *n* = 3 per group) were treated with RGD-Alg@MSC eye drops (5 μl per eye, twice daily) for 14 days. Other groups received the porous RGD-Alg microcarriers, MSCs, or no treatment under the same conditions. After treatment, mice were euthanized and important organs were removed and stained with an H&E kit. Stained sections were imaged using a Nikon digital light microscope to assess histological changes and toxicity.

### TUNEL staining

To assess potential ocular tissue damage following RGD-Alg@MSC eye drop treatment, TUNEL staining was performed on the collected eyeballs (*77*). Eyeballs were fixed in 4% PFA, embedded in paraffin, and sectioned into 5-μm slices. TUNEL staining was conducted using a TUNEL assay kit (Servicebio) according to the instructions of the manufacturer. Briefly, sections were deparaffinized, rehydrated, and treated with proteinase K (20 μg/ml) for 15 min at 37°C to permeabilize the tissue. The TUNEL reaction mixture was applied and incubated for 1 hour at 37°C in a humidified chamber. Positive controls were prepared by treating sections with deoxyribonuclease (DNase) I (100 U/ml) for 10 min to induce DNA strand breaks, while negative controls were incubated with the TUNEL reaction mixture lacking TdT. Sections were counterstained with 4′,6-diami-dino-2-phenylindole (DAPI) to label nuclei and imaged using a scanning microscope (VS200, Olympus). Apoptotic cells were identified by colocalization of TUNEL-positive signals (green) and DAPI-stained nuclei (blue).

### In vivo retention study of RGD-Alg@MSCs

To evaluate the ocular retention time of MSCs, RFP-MSCs were either encapsulated in RGD-Alg@MSCs or left as conventional suspended MSCs. For in vivo imaging, mice were briefly anesthetized with isoflurane inhalation only during the examination time points, while remaining conscious and maintaining normal activity throughout the observation period. A volume of 5 μl of eye drops containing 3 × 10^5^ RFP-MSCs was topically administered to the corneal surface of 12-week-old male ICR mice. Fluorescence imaging was performed using a multi-mode in vivo animal imaging system (AniView100) at 0, 40, 80, and 120 min post-application (*78*).

### Analysis of DCs and T_H_17 cells in the dLNs

To create a homogeneous single-cell suspension, lymph nodes were removed, mechanically cracked open, and passed through 70-μm strainers of filter paper (*32*, *79*). Using conjugated primary antibodies, this suspension was stained to identify DCs, major histocompatibility complex class II (MHC-II) [MHC-II–phycoerythrin (PE), eBioscience], CD45 (CD45-PE-CYANINE7, eBioscience), CD86 [CD86-allophycocyanin (APC), eBioscience], and CD11c [CD11c–fluorescein isothiocyanate (FITC), BioLegend]. The staining process was then prolonged for 30 min at 4°C. Using antibodies of CD45-PE-CYANINE7 (eBioscience) and CD4-FITC (BioLegend), cell suspensions were treated with a cell stimulation cocktail (BD Biosci-ences) for 5 hours at 37°C to identify T_H_17 cells. After permeabilization and fixing, cells were intracellularly labeled with PE-conjugated anti-IL-17A (IL-17A-PE, eBioscience). Isotype controls were both used. With the Accuri C6 flow cytometer and FlowJo V10 software, flow cytometric analysis was carried out. The gating rule is displayed in fig. S26.

### Immunofluorescence staining

Immunofluorescence staining was conducted according to previously established methods (*80*). In brief, mouse corneas were preserved in 4% PFA. These corneas were then treated with 10% Triton-X 100 (Beyotime) for 1 hour to permeabilize the cell membranes. To minimize nonspecific binding, corneas were treated with 5% donkey serum (Jackson ImmunoResearch) at 37°C for 1 hour. The corneas were then exposed to CD11c antibodies (1:100 dilution, PE conjugate, BioLegend) for an entire night at 4°C to measure and examine morphology. Following the incubation of the antibodies, the samples were cleaned with PBS and coated with an antifade mountant containing DAPI (Abcam). A confocal laser scanning microscope (Zeiss, Germany) was used to record the staining results, and ImageJ software was used to evaluate them. To ascertain dendritic complexity, two blinded evaluators examined enhanced magnification confocal maximum projection z-stacks, focusing on characteristic dendritic shapes and manually enumerating the dendritic tips per cell.

### Analytical statistics

GraphPad Prism (version 10) was used for all statistical analysis, and the data are shown as the mean ± SD of a minimum of three biological replicates. First, the normalcy was evaluated using the Shapiro-Wilk test. If the data were normally distributed, a repeated-measures analysis of variance was carried out for comparisons across several groups, and Dunnett’s multiple comparison test was then run. Friedman test was used to examine group differences for non-normally distributed data, with Dunn’s post hoc test used to identify which specific groups differ from each other. Adjusted *P* values were provided to reduce type I errors brought on by numerous comparisons and guarantee the reliability of the findings. When comparing just two groups, the Mann-Whitney *U* test was used for distributed non-normally data, and Welch’s *t* test was selected for regularly distributed data. Statistical significance was established as *P* < 0.05 in all situations. For the purpose of capturing potential differences in both directions, all statistical tests were two-tailed. *P* < 0.05 is denoted by *, *P* < 0.01 by **, and *P* < 0.001 by ***, while ns denotes no significance.

## References

[1] H. Shan, W. Liu, Y. Li, K. Pang, The autoimmune rheumatic disease related dry eye and its association with retinopathy. Biomolecules 13, 724 (2023). 37238594 10.3390/biom13050724 PMC10216215

[2] L. Wang, Y. Xie, Y. Deng, Prevalence of dry eye in patients with systemic lupus erythematosus: A meta-analysis. BMJ Open 11, e047081 (2021). 10.1136/bmjopen-2020-047081 PMC8483036 34588240

[3] Y. Zhang, T. Lin, A. Jiang, N. Zhao, L. Gong, Vision-related quality of life and psychological status in Chinese women with Sjogren's syndrome dry eye: A case-control study. BMC Womens Health 16, 75 (2016). 27955668 10.1186/s12905-016-0353-z PMC5154065

[4] W. Yao, Q. Le, Social-economic analysis of patients with Sjogren’s syndrome dry eye in East China: A cross-sectional study. BMC Ophthalmol. 18, 23 (2018). 29390975 10.1186/s12886-018-0694-5 PMC5796393

[5] K. S. Stewart, M. D. Abdusselamoglu, M. T. Tierney, A. Gola, Y. H. Hur, K. A. U. Gonzales, S. Yuan, A. R. Bonny, Y. Yang, N. R. Infarinato, C. J. Cowley, J. M. Levorse, H. A. Pasolli, S. Ghosh, C. V. Rothlin, E. Fuchs, Stem cells tightly regulate dead cell clearance to maintain tissue fitness. Nature 633, 407–416 (2024). 39169186 10.1038/s41586-024-07855-6 PMC11390485

[6] J. Y. Oh, R. H. Lee, Mesenchymal stromal cells for the treatment of ocular autoimmune diseases. Prog. Retin. Eye Res. 85, 100967 (2021). 33775824 10.1016/j.preteyeres.2021.100967 PMC8922475

[7] L. Chen, Z. Jin, W. Feng, L. Sun, H. Xu, C. Wang, A hyperelastic hydrogel with an ultralarge reversible biaxial strain. Science 383, 1455–1461 (2024). 38547271 10.1126/science.adh3632

[8] P. Li, W. Sun, J. Li, J. P. Chen, X. Wang, Z. Mei, G. Jin, Y. Lei, R. Xin, M. Yang, J. Xu, X. Pan, C. Song, X. Y. Deng, X. Lei, K. Liu, X. Wang, Y. Zheng, J. Zhu, S. Lv, Z. Zhang, X. Dai, T. Lei, N-type semiconducting hydrogel. Science 384, 557–563 (2024). 38696573 10.1126/science.adj4397

[9] A. P. Dhand, M. D. Davidson, H. M. Zlotnick, T. J. Kolibaba, J. P. Killgore, J. A. Burdick, Additive manufacturing of highly entangled polymer networks. Science 385, 566–572 (2024). 39088628 10.1126/science.adn6925 PMC11921614

[10] J. M. Kronenfeld, L. Rother, M. A. Saccone, M. T. Dulay, J. M. DeSimone, Roll-to-roll, high-resolution 3D printing of shape-specific particles. Nature 627, 306–312 (2024). 38480965 10.1038/s41586-024-07061-4 PMC10937373

[11] W. Xue, D. Lee, Y. Kong, M. Kuss, Y. Huang, T. Kim, S. Chung, A. T. Dudley, S. H. Ro, B. Duan, A facile strategy for the fabrication of cell-laden porous alginate hydrogels based on two-phase aqueous emulsions. Adv. Funct. Mater. 33, 2214129 (2023). 38131003 10.1002/adfm.202214129 PMC10732541

[12] W. Sun, J. Zhang, Y. Qin, H. Tang, Y. Chen, W. Lin, Y. She, K. Zhang, J. Yin, C. Chen, A simple and efficient strategy for preparing a cell-spheroid-based bioink. Adv. Healthc. Mater. 11, e2200648 (2022). 35543489 10.1002/adhm.202200648

[13] J. M. Montanero, A. M. Gañán-Calvo, Dripping, jetting and tip streaming. Rep. Prog. Phys. 83, 097001 (2020). 32647097 10.1088/1361-6633/aba482

[14] L. Xuan, Y. Hou, L. Liang, J. Wu, K. Fan, L. Lian, J. Qiu, Y. Miao, H. Ravanbakhsh, M. Xu, G. Tang, Microgels for cell delivery in tissue engineering and regenerative medicine. Nanomicro Lett. 16, 218 (2024). 38884868 10.1007/s40820-024-01421-5 PMC11183039

[15] J. A. Rowley, G. Madlambayan, D. J. Mooney, Alginate hydrogels as synthetic extracellular matrix materials. Biomaterials 20, 45–53 (1999). 9916770 10.1016/s0142-9612(98)00107-0

[16] W. Kapadia, N. Qin, P. Zhao, C. M. Phan, L. Haines, L. Jones, C. L. Ren, Shear-thinning and temperature-dependent viscosity relationships of contemporary ocular lubricants. Transl. Vis. Sci. Technol. 11, 1 (2022). 10.1167/tvst.11.3.1 PMC8899858 35234832

[17] A. H. A. Mohamed-Ahmed, D. Kuguminkiriza, Local production of eye drops in the hospital or pharmacy setting: Considerations and safety tips. Community Eye Health 36, 17–18 (2023). PMC10236422 37273803

[18] A. Uccelli, L. Moretta, V. Pistoia, Mesenchymal stem cells in health and disease. Nat. Rev. Immunol. 8, 726–736 (2008). 19172693 10.1038/nri2395

[19] P. Versura, V. Profazio, C. Schiavi, E. C. Campos, Hyperosmolar stress upregulates HLA-DR expression in human conjunctival epithelium in dry eye patients and in vitro models. Invest. Ophthalmol. Vis. Sci. 52, 5488–5496 (2011). 21498621 10.1167/iovs.11-7215

[20] Y. Xia, Y. Zhang, Y. Du, Z. Wang, L. Cheng, Z. Du, Comprehensive dry eye therapy: Overcoming ocular surface barrier and combating inflammation, oxidation, and mitochondrial damage. J. Nanobiotechnology 22, 233 (2024). 38725011 10.1186/s12951-024-02503-7 PMC11080212

[21] F. M. Yang, D. Fan, X. Q. Yang, F. H. Zhu, M. J. Shao, Q. Li, Y. T. Liu, Z. M. Lin, S. Q. Cao, W. Tang, S. J. He, J. P. Zuo, The artemisinin analog SM934 alleviates dry eye disease in rodent models by regulating TLR4/NF-κB/NLRP3 signaling. Acta Pharmacol. Sin. 42, 593–603 (2021). 32747720 10.1038/s41401-020-0484-5 PMC8114933

[22] Y. Shi, G. Hu, J. Su, W. Li, Q. Chen, P. Shou, C. Xu, X. Chen, Y. Huang, Z. Zhu, X. Huang, X. Han, N. Xie, G. Ren, Mesenchymal stem cells: A new strategy for immunosuppression and tissue repair. Cell Res. 20, 510–518 (2010). 20368733 10.1038/cr.2010.44

[23] G. Wang, K. Cao, K. Liu, Y. Xue, A. I. Roberts, F. Li, Y. Han, A. B. Rabson, Y. Wang, Y. Shi, Kynurenic acid, an IDO metabolite, controls TSG-6-mediated immunosuppression of human mesenchymal stem cells. Cell Death Differ. 25, 1209–1223 (2018). 29238069 10.1038/s41418-017-0006-2 PMC6030103

[24] S. W. Yun, Y. H. Son, D. Y. Lee, Y. J. Shin, M. J. Han, D. H. Kim, Lactobacillus plantarum and Bifidobacterium bifidum alleviate dry eye in mice with exorbital lacrimal gland excision by modulating gut inflammation and microbiota. Food Funct. 12, 2489–2497 (2021). 33656499 10.1039/d0fo02984j

[25] S. H. Yang, M. J. Park, I. H. Yoon, S. Y. Kim, S. H. Hong, J. Y. Shin, H. Y. Nam, Y. H. Kim, B. Kim, C. G. Park, Soluble mediators from mesenchymal stem cells suppress T cell proliferation by inducing IL-10. Exp. Mol. Med. 41, 315–324 (2009). 19307751 10.3858/emm.2009.41.5.035 PMC2701980

[26] B. Wang, W. Liu, J. J. Li, S. Chai, D. Xing, H. Yu, Y. Zhang, W. Yan, Z. Xu, B. Zhao, Y. Du, Q. Jiang, A low dose cell therapy system for treating osteoarthritis: In vivo study and in vitro mechanistic investigations. Bioact. Mater. 7, 478–490 (2022). 34466747 10.1016/j.bioactmat.2021.05.029 PMC8379370

[27] Y. H. Kim, Z. R. Li, L. Cui, Y. Li, H. J. Yoon, W. Choi, J. B. Lee, Z. G. Liu, K. C. Yoon, Expression of Nod-like receptors and clinical correlations in patients with dry eye disease. Am. J. Ophthalmol. 200, 150–160 (2019). 30653959 10.1016/j.ajo.2019.01.002

[28] H. Guo, Y. Ju, M. Choi, M. C. Edman, S. G. Louie, S. F. Hamm-Alvarez, J. A. MacKay, Supra-lacrimal protein-based carriers for cyclosporine A reduce Th17-mediated autoimmunity in murine model of Sjögren's syndrome. Biomaterials 283, 121441 (2022). 35306230 10.1016/j.biomaterials.2022.121441 PMC8982551

[29] I. Miletich, Molecular regulation of ocular gland development. Semin. Cell Dev. Biol. 91, 66–74 (2019). 30266427 10.1016/j.semcdb.2018.07.023

[30] H. Zhu, F. Yang, B. Tang, X. M. Li, Y. N. Chu, Y. L. Liu, S. G. Wang, D. C. Wu, Y. Zhang, Mesenchymal stem cells attenuated PLGA-induced inflammatory responses by inhibiting host DC maturation and function. Biomaterials 53, 688–698 (2015). 25890764 10.1016/j.biomaterials.2015.03.005

[31] Y. Chen, R. Dana, Autoimmunity in dry eye disease—An updated review of evidence on effector and memory Th17 cells in disease pathogenicity. Autoimmun. Rev. 20, 102933 (2021). 34509656 10.1016/j.autrev.2021.102933 PMC8530974

[32] C. Yu, P. Chen, J. Xu, S. Wei, Q. Cao, C. Guo, X. Wu, G. Di, Corneal epithelium-derived netrin-1 alleviates dry eye disease via regulating dendritic cell activation. Invest. Ophthalmol. Vis. Sci. 63, 1 (2022). 10.1167/iovs.63.6.1 PMC9172049 35648640

[33] X. Tan, Y. Chen, W. Foulsham, A. Amouzegar, T. Inomata, Y. Liu, S. K. Chauhan, R. Dana, The immunoregulatory role of corneal epithelium-derived thrombospondin-1 in dry eye disease. Ocul. Surf. 16, 470–477 (2018). 30055331 10.1016/j.jtos.2018.07.005 PMC6289260

[34] H. Levine, J. Hwang, H. Dermer, D. Mehra, W. Feuer, A. Galor, Relationships between activated dendritic cells and dry eye symptoms and signs. Ocul. Surf. 21, 186–192 (2021). 34102312 10.1016/j.jtos.2021.06.001 PMC8328957

[35] D. Lucchesi, R. Coleby, E. Pontarini, E. Prediletto, F. Rivellese, D. G. Hill, A. Derrac Soria, S. A. Jones, I. R. Humphreys, N. Sutcliffe, A. R. Tappuni, C. Pitzalis, G. W. Jones, M. Bombardieri, Impaired interleukin-27-mediated control of CD4^+^ T cell function impact on ectopic lymphoid structure formation in patients with Sjögren's syndrome. Arthritis Rheumatol. 72, 1559–1570 (2020). 32307922 10.1002/art.41289

[36] L. Geng, X. Tang, K. Zhou, D. Wang, S. Wang, G. Yao, W. Chen, X. Gao, W. Chen, S. Shi, N. Shen, X. Feng, L. Sun, MicroRNA-663 induces immune dysregulation by inhibiting TGF-β1 production in bone marrow-derived mesenchymal stem cells in patients with systemic lupus erythematosus. Cell. Mol. Immunol. 16, 260–274 (2019). 30886422 10.1038/cmi.2018.1 PMC6460486

[37] G. Yao, J. Qi, J. Liang, B. Shi, W. Chen, W. Li, X. Tang, D. Wang, L. Lu, W. Chen, S. Shi, Y. Hou, L. Sun, Mesenchymal stem cell transplantation alleviates experimental Sjögren's syndrome through IFN-β/IL-27 signaling axis. Theranostics 9, 8253–8265 (2019). 31754394 10.7150/thno.37351 PMC6857067

[38] Y. Sun, W. Deng, G. Yao, W. Chen, X. Tang, X. Feng, L. Lu, L. Sun, Citrullinated fibrinogen impairs immunomodulatory function of bone marrow mesenchymal stem cells by triggering toll-like receptor. Clin. Immunol. 193, 38–45 (2018). 29373844 10.1016/j.clim.2018.01.008

[39] X. Wu, H. Zhu, J. Che, Y. Xu, Q. Tan, Y. Zhao, Stem cell niche-inspired microcarriers with ADSCs encapsulation for diabetic wound treatment. Bioact. Mater. 26, 159–168 (2023). 36923266 10.1016/j.bioactmat.2023.02.031 PMC10008968

[40] C. K. Kuo, P. X. Ma, Ionically crosslinked alginate hydrogels as scaffolds for tissue engineering: Part 1. Structure, gelation rate and mechanical properties. Biomaterials 22, 511–521 (2001). 11219714 10.1016/s0142-9612(00)00201-5

[41] D. Huang, J. Wang, M. Nie, G. Chen, Y. Zhao, Pollen-inspired adhesive multilobe microparticles from microfluidics for intestinal drug delivery. Adv. Mater. 35, e2301192 (2023). 37004147 10.1002/adma.202301192

[42] N. C. Hunt, D. Hallam, A. Karimi, C. B. Mellough, J. Chen, D. H. W. Steel, M. Lako, 3D culture of human pluripotent stem cells in RGD-alginate hydrogel improves retinal tissue development. Acta Biomater. 49, 329–343 (2017). 27826002 10.1016/j.actbio.2016.11.016

[43] J. Jang, Y.-J. Seol, H. J. Kim, J. Kundu, S. W. Kim, D.-W. Cho, Effects of alginate hydrogel cross-linking density on mechanical and biological behaviors for tissue engineering. J. Mech. Behav. Biomed. Mater. 37, 69–77 (2014). 24880568 10.1016/j.jmbbm.2014.05.004

[44] F. C. Kung, Injectable collagen/RGD systems for bone tissue engineering applications. Biomed. Mater. Eng. 29, 241–251 (2018). 29457597 10.3233/BME-171726

[45] K. Shen, Z. Lv, Y. Yang, H. Wang, J. Liu, Q. Chen, Z. Liu, M. Zhang, J. Liu, Y. Cheng, A wet-adhesion and swelling-resistant hydrogel for fast hemostasis, accelerated tissue injury healing and bioelectronics. Adv. Mater. 37, e2414092 (2025). 39713944 10.1002/adma.202414092

[46] M. A. Kinney, T. A. Hookway, Y. Wang, T. C. McDevitt, Engineering three-dimensional stem cell morphogenesis for the development of tissue models and scalable regenerative therapeutics. Ann. Biomed. Eng. 42, 352–367 (2014). 24297495 10.1007/s10439-013-0953-9 PMC3939035

[47] K. Kim, J. Cheng, Q. Liu, X. Y. Wu, Y. Sun, Investigation of mechanical properties of soft hydrogel microcapsules in relation to protein delivery using a MEMS force sensor. J. Biomed. Mater. Res. A 92, 103–113 (2010). 19165782 10.1002/jbm.a.32338

[48] G. Huerta-Ángeles, F. Ondreáš, M. Brandejsová, K. Kopecká, H. Vagnerová, J. Kulhánek, T. Drmota, Formulation of hyaluronan grafted with dodecanoic acid as a potential ophthalmic treatment. Carbohydr. Polym. 246, 116578 (2020). 32747245 10.1016/j.carbpol.2020.116578

[49] M. Yang, T. Chen, X. Chen, H. Pan, G. Zhao, Z. Chen, N. Zhao, Q. Ye, M. Chen, S. Zhang, R. Gao, K. M. Meek, S. Hayes, X. Ma, X. Li, Y. Wu, Y. Zhang, N. Kong, W. Tao, X. Zhou, J. Huang, Development of graphitic carbon nitride quantum dots-based oxygen self-sufficient platforms for enhanced corneal crosslinking. Nat. Commun. 15, 5508 (2024). 38951161 10.1038/s41467-024-49645-8 PMC11217369

[50] W. Zhang, L. Cai, J. Gan, Y. Zhao, Photothermal responsive porous hollow microneedles as Chinese medicine versatile delivery system for wound healing. Smart Med. 3, e20240007 (2024). 39420949 10.1002/SMMD.20240007 PMC11425051

[51] G. Chen, F. Wang, M. Nie, H. Zhang, H. Zhang, Y. Zhao, Roe-inspired stem cell microcapsules for inflammatory bowel disease treatment. Proc. Natl. Acad. Sci. U.S.A. 118, e2112704118 (2021). 34686606 10.1073/pnas.2112704118 PMC8639345

[52] M. D. Darrabie, W. F. Kendall Jr., E. C. Opara, Characteristics of Poly-L-Ornithine-coated alginate microcapsules. Biomaterials 26, 6846–6852 (2005). 15955558 10.1016/j.biomaterials.2005.05.009

[53] J. Yan, F. Chen, B. G. Amsden, Cell sheets prepared via gel-sol transition of calcium RGD-alginate. Acta Biomater. 30, 277–284 (2016). 26537201 10.1016/j.actbio.2015.10.046

[54] S. Viswanathan, Y. Shi, J. Galipeau, M. Krampera, K. Leblanc, I. Martin, J. Nolta, D. G. Phinney, L. Sensebe, Mesenchymal stem versus stromal cells: International Society for Cell & Gene Therapy (ISCT®) Mesenchymal Stromal Cell committee position statement on nomenclature. Cytotherapy 21, 1019–1024 (2019). 31526643 10.1016/j.jcyt.2019.08.002

[55] Y. B. Ji, S. Lee, H. J. Ju, H. E. Kim, J. H. Noh, S. Choi, K. Park, H. B. Lee, M. S. Kim, Preparation and evaluation of injectable microsphere formulation for longer sustained release of donepezil. J. Control. Release 356, 43–58 (2023). 36841288 10.1016/j.jconrel.2023.02.024

[56] X. Wang, C. Yang, Y. Yu, Y. Zhao, In situ 3D bioprinting living photosynthetic scaffolds for autotrophic wound healing. Research 2022, 9794745 (2022). 35387266 10.34133/2022/9794745 PMC8961369

[57] S. Li, Z. Lu, Y. Huang, Y. Wang, Q. Jin, X. Shentu, J. Ye, J. Ji, K. Yao, H. Han, Anti-oxidative and anti-inflammatory micelles: Break the dry eye vicious cycle. Adv. Sci. 9, e2200435 (2022). 10.1002/advs.202200435 PMC9189644 35435328

[58] J. M. Sierra-Parraga, A. Merino, M. Eijken, H. Leuvenink, R. Ploeg, B. K. Møller, B. Jespersen, C. C. Baan, M. J. Hoogduijn, Reparative effect of mesenchymal stromal cells on endothelial cells after hypoxic and inflammatory injury. Stem Cell Res. Ther. 11, 352 (2020). 32787906 10.1186/s13287-020-01869-3 PMC7424997

[59] X. Liu, Z. Chen, J. Bai, X. Li, X. Chen, Z. Li, H. Pan, S. Li, Q. Gao, N. Zhao, A. Chen, H. Xu, Y. Wen, L. Du, M. Yang, X. Zhou, J. Huang, Multifunctional hydrogel eye drops for synergistic treatment of ocular inflammatory disease. ACS Nano 17, 25377–25390 (2023). 37890030 10.1021/acsnano.3c08869

[60] M. J. Lee, D. H. Kim, J. S. Ryu, A. Y. Ko, J. H. Ko, M. K. Kim, W. R. Wee, S. I. Khwarg, J. Y. Oh, Topical TSG-6 administration protects the ocular surface in two mouse models of inflammation-related dry eye. Invest. Ophthalmol. Vis. Sci. 56, 5175–5181 (2015). 26244293 10.1167/iovs.14-16307

[61] J. Y. Oh, J. S. Ryu, H. J. Kim, N. Kouvatsos, R. J. Dodd, S. H. Choi, Y. J. Kim, C. M. Milner, A. J. Day, The Link module of human TSG-6 (Link_TSG6) promotes wound healing, suppresses inflammation and improves glandular function in mouse models of dry eye disease. Ocul. Surf. 24, 40–50 (2022). 34968766 10.1016/j.jtos.2021.12.012

[62] Y. Li, D. Zhang, L. Xu, L. Dong, J. Zheng, Y. Lin, J. Huang, Y. Zhang, Y. Tao, X. Zang, D. Li, M. Du, Cell-cell contact with proinflammatory macrophages enhances the immunotherapeutic effect of mesenchymal stem cells in two abortion models. Cell. Mol. Immunol. 16, 908–920 (2019). 30778166 10.1038/s41423-019-0204-6 PMC6884632

[63] S. Ghannam, J. Pène, G. Moquet-Torcy, C. Jorgensen, H. Yssel, Mesenchymal stem cells inhibit human Th17 cell differentiation and function and induce a T regulatory cell phenotype. J. Immunol. 185, 302–312 (2010). 20511548 10.4049/jimmunol.0902007

[64] R. Liu, D. Su, M. Zhou, X. Feng, X. Li, L. Sun, Umbilical cord mesenchymal stem cells inhibit the differentiation of circulating T follicular helper cells in patients with primary Sjögren's syndrome through the secretion of indoleamine 2,3-dioxygenase. Rheumatology 54, 332–342 (2015). 25169988 10.1093/rheumatology/keu316

[65] W. X. Gao, Y. Q. Sun, J. Shi, C. L. Li, S. B. Fang, D. Wang, X. Q. Deng, W. Wen, Q. L. Fu, Effects of mesenchymal stem cells from human induced pluripotent stem cells on differentiation, maturation, and function of dendritic cells. Stem Cell Res. Ther. 8, 48 (2017). 28253916 10.1186/s13287-017-0499-0 PMC5333407

[66] G. M. Spaggiari, H. Abdelrazik, F. Becchetti, L. Moretta, MSCs inhibit monocyte-derived DC maturation and function by selectively interfering with the generation of immature DCs: Central role of MSC-derived prostaglandin E2. Blood 113, 6576–6583 (2009). 19398717 10.1182/blood-2009-02-203943

[67] L. Liang, C. Kong, J. Li, G. Liu, J. Wei, G. Wang, Q. Wang, Y. Yang, D. Shi, X. Li, Y. Ma, Distinct microbes, metabolites, and the host genome define the multi-omics profiles in right-sided and left-sided colon cancer. Microbiome 12, 274 (2024). 39731152 10.1186/s40168-024-01987-7 PMC11681701

[68] D. Kim, J. M. Paggi, C. Park, C. Bennett, S. L. Salzberg, Graph-based genome alignment and genotyping with HISAT2 and HISAT-genotype. Nat. Biotechnol. 37, 907–915 (2019). 31375807 10.1038/s41587-019-0201-4 PMC7605509

[69] M. Pertea, G. M. Pertea, C. M. Antonescu, T. C. Chang, J. T. Mendell, S. L. Salzberg, StringTie enables improved reconstruction of a transcriptome from RNA-seq reads. Nat. Biotechnol. 33, 290–295 (2015). 25690850 10.1038/nbt.3122 PMC4643835

[70] Y. Chen, L. Chen, A. T. L. Lun, P. L. Baldoni, G. K., edgeR v4: Powerful differential analysis of sequencing data with expanded functionality and improved support for small counts and larger datasets. Nucleic Acids Res. 53, gkaf018 (2025). 39844453 10.1093/nar/gkaf018 PMC11754124

[71] M. Ashburner, C. A. Ball, J. A. Blake, D. Botstein, H. Butler, J. M. Cherry, A. P. Davis, K. Dolinski, S. S. Dwight, J. T. Eppig, M. A. Harris, D. P. Hill, L. Issel-Tarver, A. Kasarskis, S. Lewis, J. C. Matese, J. E. Richardson, M. Ringwald, G. M. Rubin, G. Sherlock, Gene ontology: Tool for the unification of biology. The Gene Ontology Consortium. Nat. Genet. 25, 25–29 (2000). 10802651 10.1038/75556 PMC3037419

[72] M. Kanehisa, M. Furumichi, Y. Sato, M. Ishiguro-Watanabe, M. Tanabe, KEGG: Integrating viruses and cellular organisms. Nucleic Acids Res. 49, D545–D551 (2021). 33125081 10.1093/nar/gkaa970 PMC7779016

[73] X. Zheng, C. S. de Paiva, D. Q. Li, W. J. Farley, S. C. Pflugfelder, Desiccating stress promotion of Th17 differentiation by ocular surface tissues through a dendritic cell-mediated pathway. Invest. Ophthalmol. Vis. Sci. 51, 3083–3091 (2010). 20130281 10.1167/iovs.09-3838 PMC2891467

[74] S. R. da Costa, K. Wu, M. M. Veigh, M. Pidgeon, C. Ding, J. E. Schechter, S. F. Hamm-Alvarez, Male NOD mouse external lacrimal glands exhibit profound changes in the exocytotic pathway early in postnatal development. Exp. Eye Res. 82, 33–45 (2006). 16005870 10.1016/j.exer.2005.04.019 PMC1351294

[75] A. Lemp, Report of the National Eye Institute/Industry workshop on clinical trials in dry eyes. Eye Contact Lens 21, 221–232 (1995). 8565190

[76] B. Puentes, E. A. Hisey, M. Ferneding, V. N. Ureno, M. A. H. Do, P. M. Karpinen, C. C. Luo, S. M. Thomasy, B. C. Leonard, Development and validation of a method to generate phenol red thread tests. Ocul. Surf. 34, 262–266 (2024). 39127389 10.1016/j.jtos.2024.08.007 PMC11913062

[77] H. Gao, M. Chen, Y. Liu, D. Zhang, J. Shen, N. Ni, Z. Tang, Y. Ju, X. Dai, A. Zhuang, Z. Wang, Q. Chen, X. Fan, Z. Liu, P. Gu, Injectable anti-inflammatory supramolecular nanofiber hydrogel to promote anti-VEGF therapy in age-related macular degeneration treatment. Adv. Mater. 35, e2204994 (2023). 36349821 10.1002/adma.202204994

[78] X. Yang, B. Wang, H. Zeng, L. Liang, R. Zhang, W. Deng, X. Zhao, J. Yuan, A modified polydopamine nanoparticle loaded with melatonin for synergistic ROS scavenging and anti-inflammatory effects in the treatment of dry eye disease. Adv. Healthc. Mater. 14, e2404372 (2025). 39828670 10.1002/adhm.202404372

[79] R. B. Singh, T. Blanco, S. K. Mittal, Y. Taketani, S. K. Chauhan, Y. Chen, R. Dana, Pigment Epithelium-derived Factor secreted by corneal epithelial cells regulates dendritic cell maturation in dry eye disease. Ocul. Surf. 18, 460–469 (2020). 32387568 10.1016/j.jtos.2020.05.002 PMC7322788

[80] K. Senthil, H. Jiao, L. E. Downie, H. R. Chinnery, Altered corneal epithelial dendritic cell morphology and phenotype following acute exposure to hyperosmolar saline. Invest. Ophthalmol. Vis. Sci. 62, 38 (2021). 10.1167/iovs.62.2.38 PMC7910639 33625479

