# Supplementary material for: MSC-encapsulated porous microparticle eye drops for autoimmune dry eye disease treatment in NOD mice
Source: Sci Adv. 2025 Sep 24;11(39):eadu9772. doi: 10.1126/sciadv.adu9772 (PMC12459467; doi:10.1126/sciadv.adu9772)
Supplement: Supplementary file 1 — Figs. S1 to S26 Tables S1 and S2 [file sciadv.adu9772_sm.pdf]

Science Advances
AAAS

Supplementary Materials for

MSC-encapsulated porous microparticle eye drops for autoimmune dry eye disease treatment in NOD mice

Taige Chen *et al.*

Corresponding author: Xuebing Feng, fengxuebing@hotmail.com; Bin Kong, kongbin13@szu.edu.cn; Yuanjin Zhao, yjzhao@seu.edu.cn

Sci. Adv. **11**, eadu9772 (2025)
DOI: 10.1126/sciadv.adu9772

This PDF file includes:

Figs. S1 to S26
Tables S1 and S2

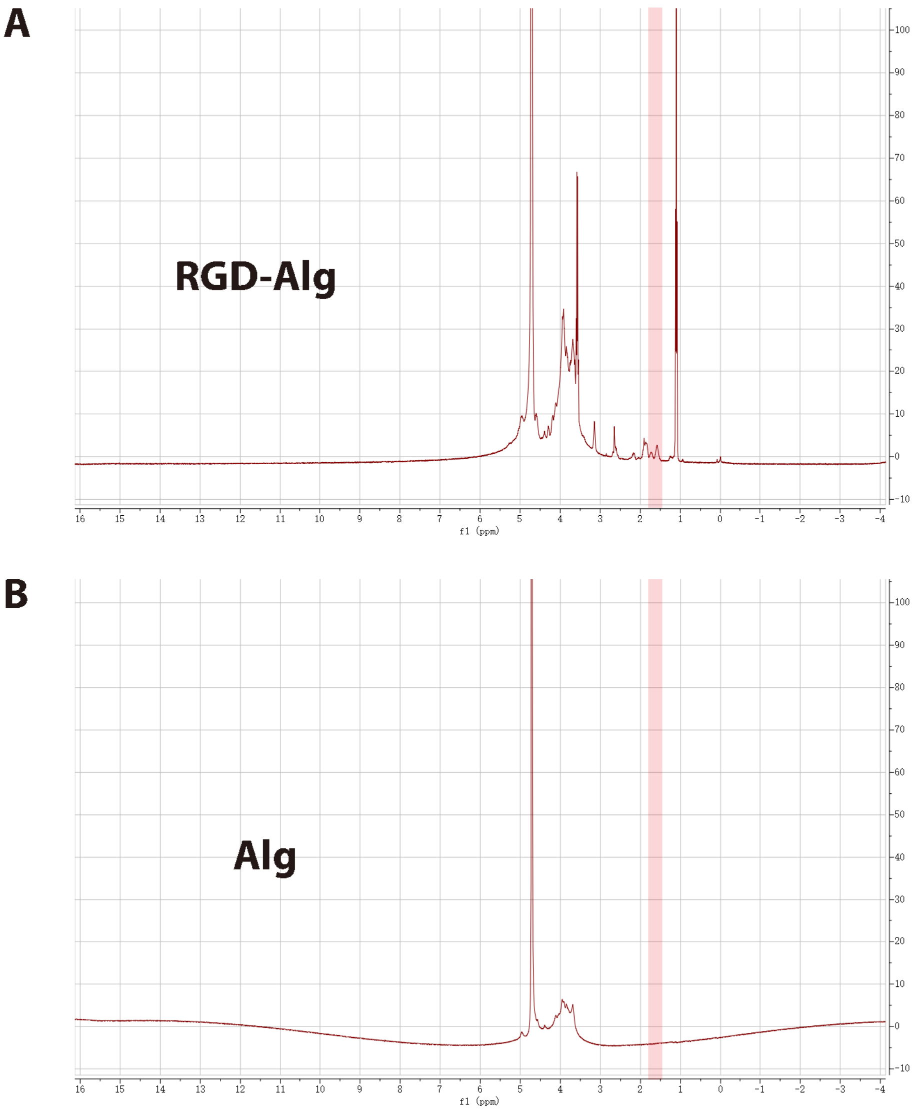

Figure S1. Full ¹H NMR spectrum of RGD-Alg (A) and Alg (B). The RGD-modified alginate showed proton peaks corresponding to arginine residues at 1.4–1.8 ppm.

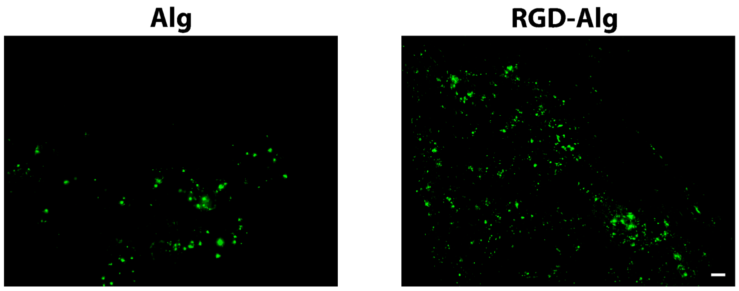

Figure S2. Images of cell adhesion experiments on Alginate hydrogel and RGD-Alg hydrogel.

The scale bar is 100 μm.

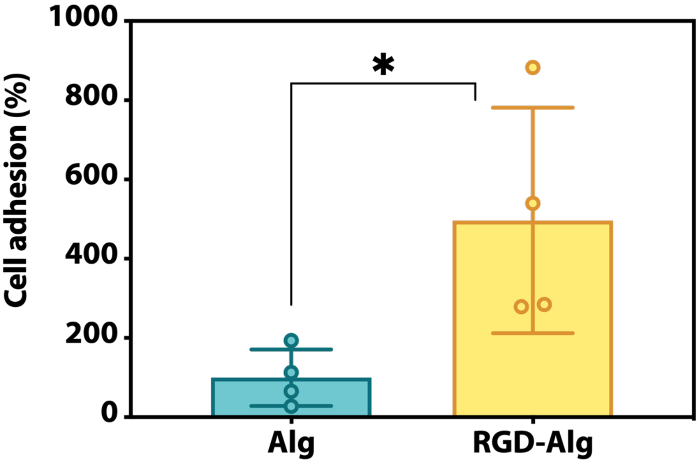

Figure S3. Quantification of cells after the cell adhesion experiments on Alginate hydrogel and RGD-Alg hydrogel.

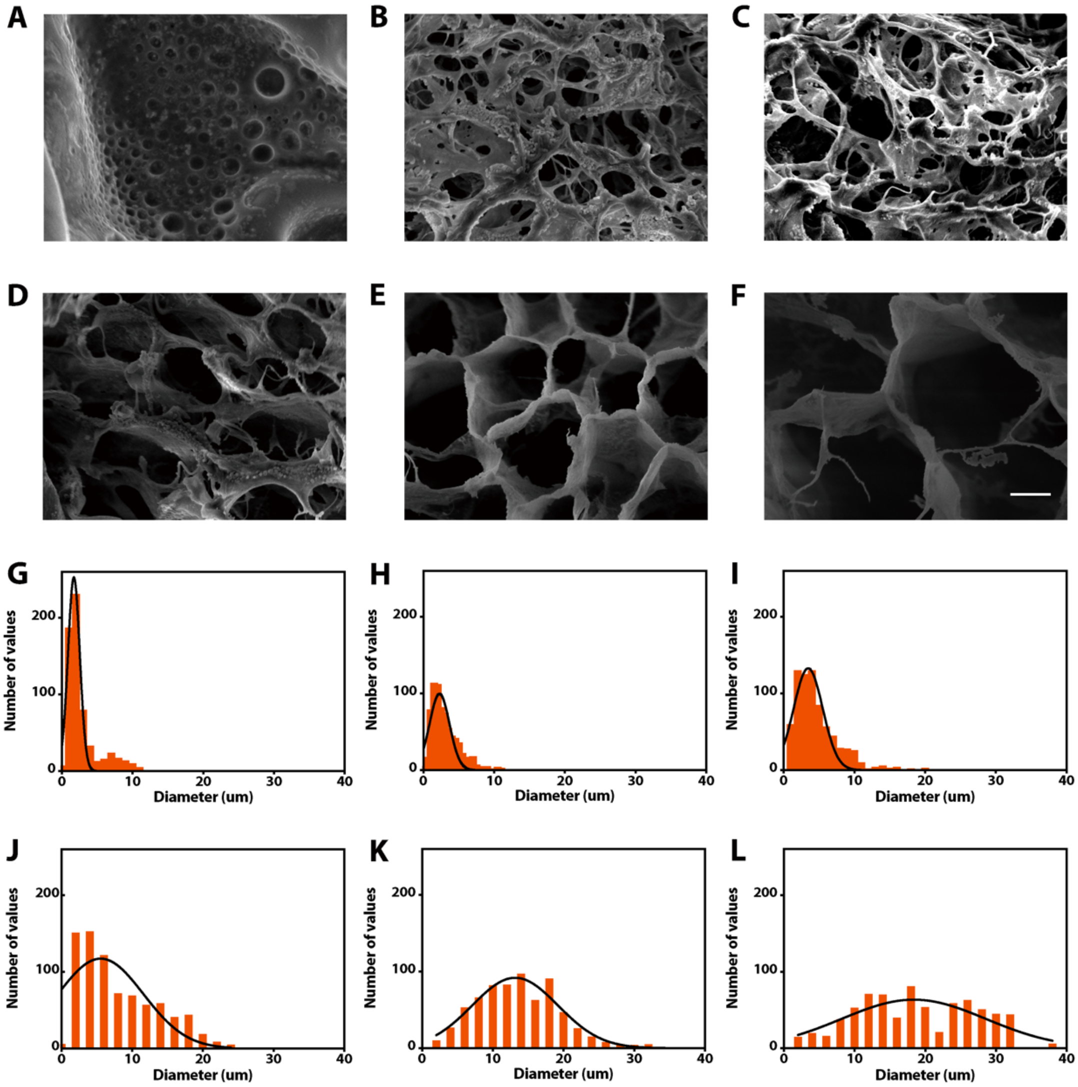

Figure S4. Microstructure of porous RGD-Alg hydrogel. SEM shows the microstructure of

porous RGD-Alg hydrogels with RGD-Alg concentration of 2% and PEO concentrations of 0.01% (A), 0.05% (B), 0.1% (C), 0.5% (D), 1% (E), and 1.5% (F). Scale bar is 10 μm. Statistical diagram of the diameter distribution of hydrogel pores at different PEO concentrations (g-l).

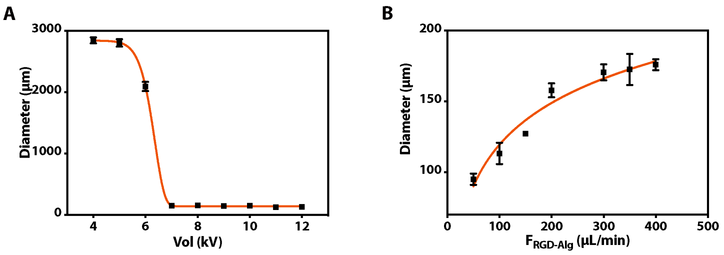

Figure S5.The influences of voltage (A), flow rate of RGD-Alg pre-gel (B) and RGD-Alg concentration (C) on microcarrier diameter were detected and summarized.

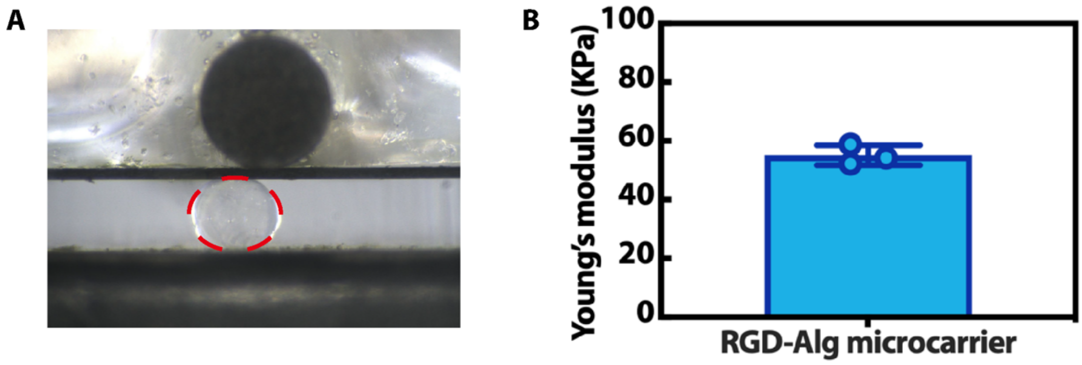

Figure S6. Micron-scale mechanical test of the porous RGD-Alg microcarriers. (A) The testing process of the micron-scale mechanical testing system, with the red circle indicating the microcarrier being tested. (B) Young's modulus (KPa) of the porous RGD-Alg microcarriers.

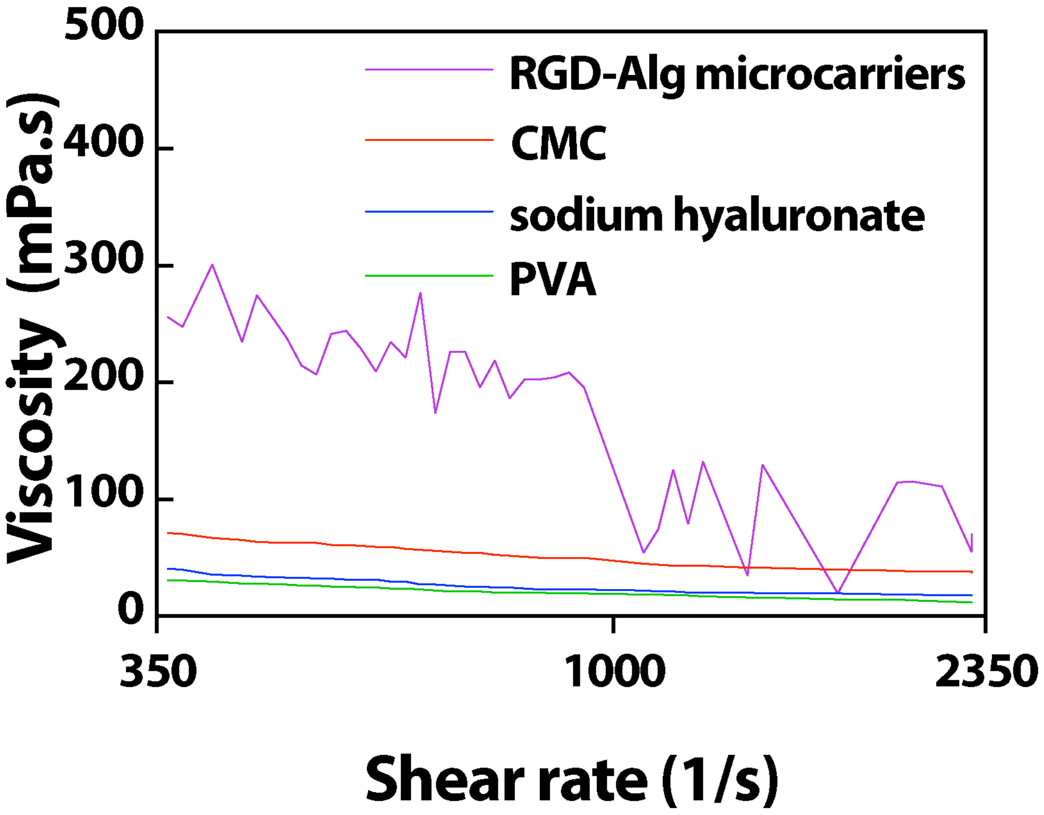

Figure S7. Rheological properties of the porous RGD-Alg microcarriers. Viscosity (η) as a function of shear rate (350-2350 s^{-1}) for the porous RGD-Alg microcarriers, 1.5% polyvinyl alcohol (PVA), 0.3% sodium hyaluronate and 0.5% sodium carboxymethyl cellulose (CMC) solutions .

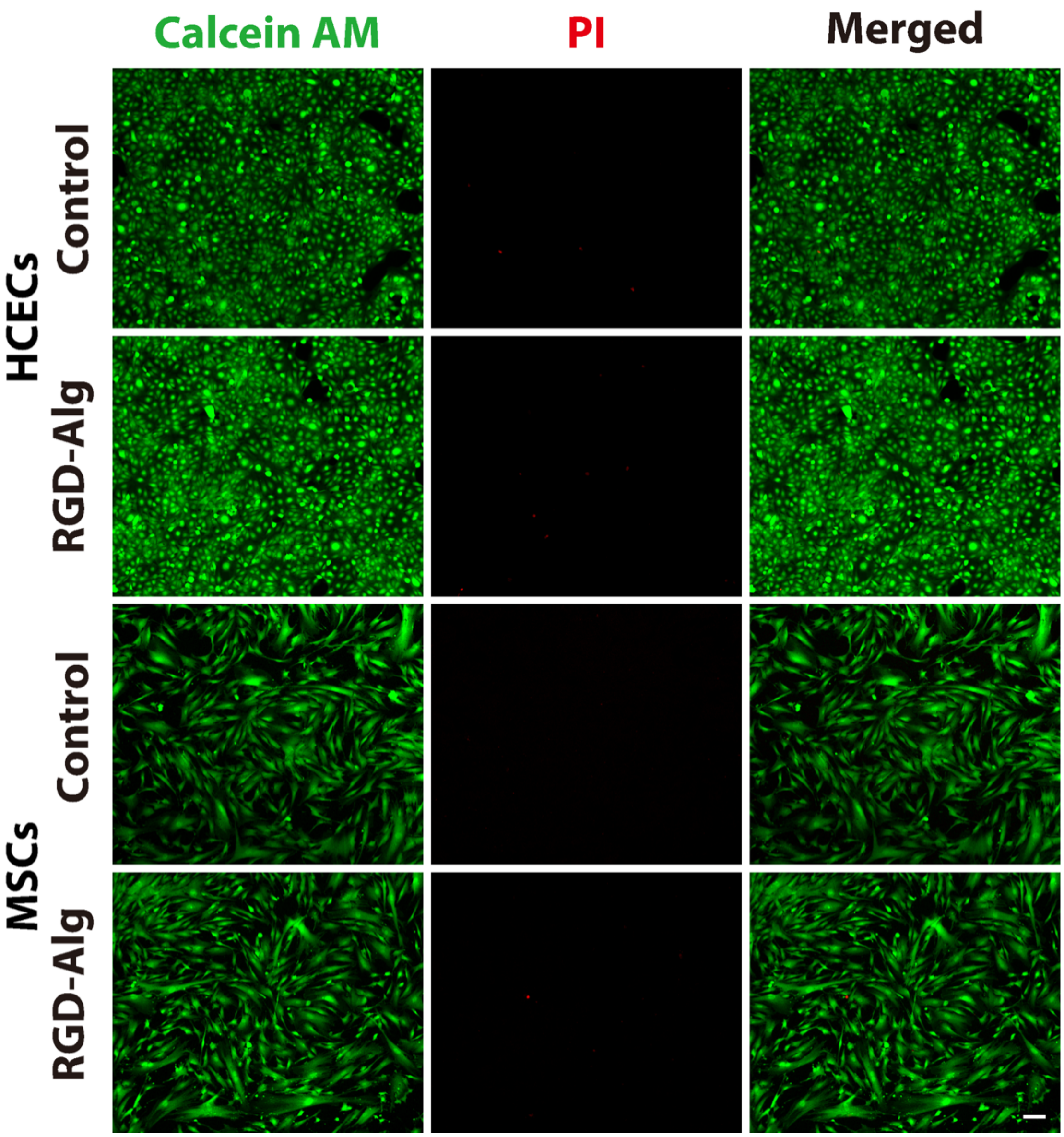

Figure S8. Representative live/dead photos of HCECs (A) and MSCs (B) following a 24-hour exposure to the porous RGD-Alg microcarriers leaching solution. Red cells are dead; green cells are living. There is a 100 μm scale bar.

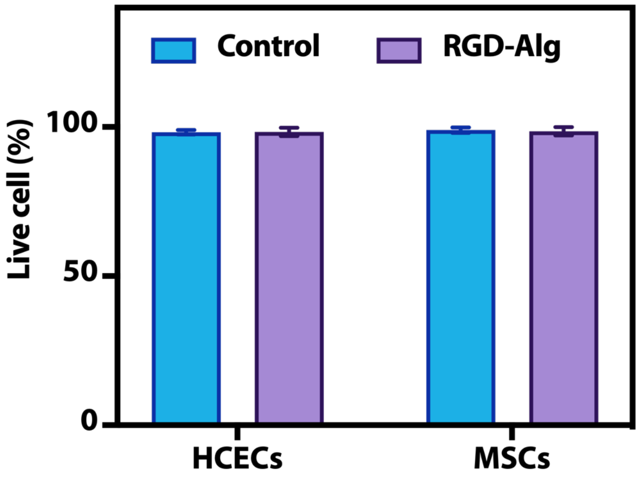

Figure S9. **Quantification of live cells following a 24-hour exposure to the porous RGD-Alg microcarriers leaching solution.**

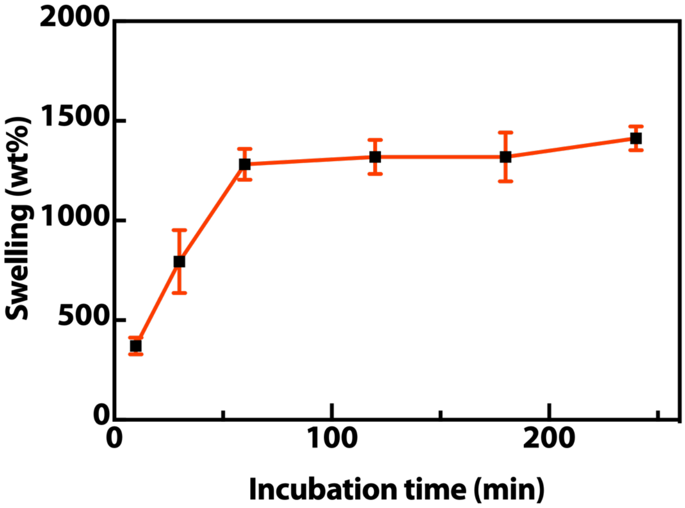

Figure S10. The swelling ratio of the porous RGD-Alg microcarriers.

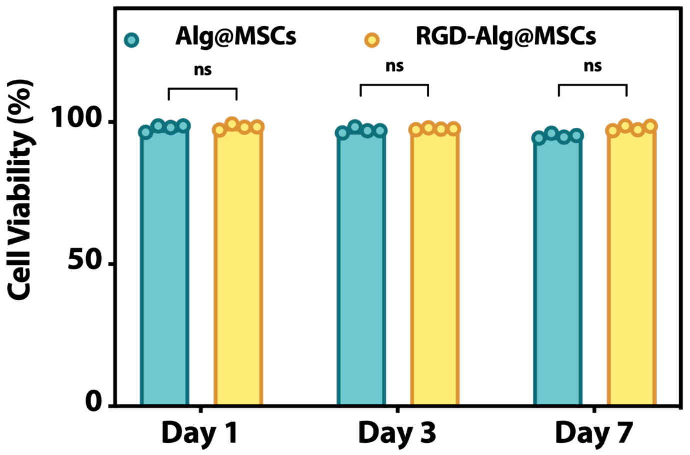

Figure S11. The cell viability of MSCs in the porous Alg microcarriers and the porous RGD-Alg microcarriers.

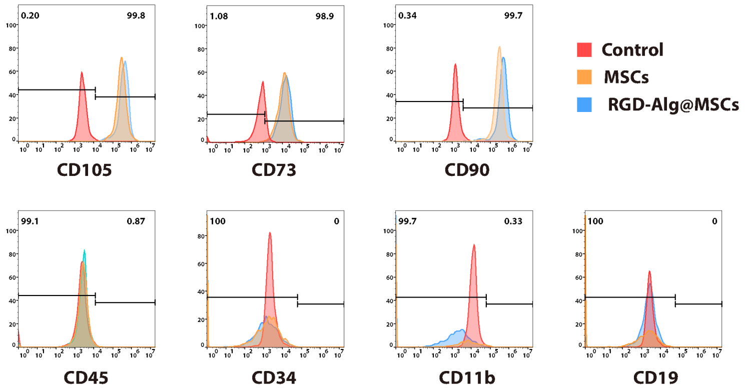

Figure S12. Flow cytometry analysis was used to identify human umbilical cord-derived MSCs and the cell stemness of MSCs encapsulated in RGD-Alg microparticles based on the expression of particular surface markers.

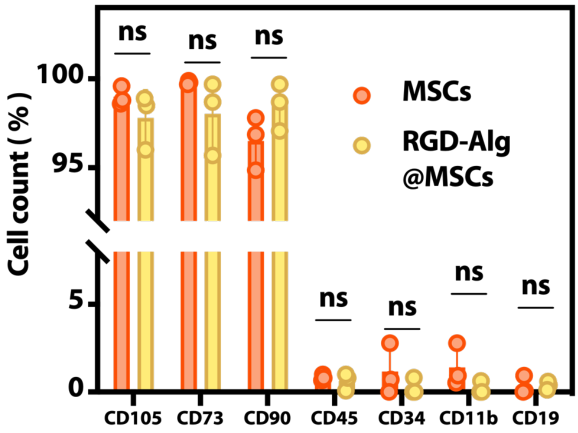

Figure S13. Quantitative analysis of the positive cell populations.

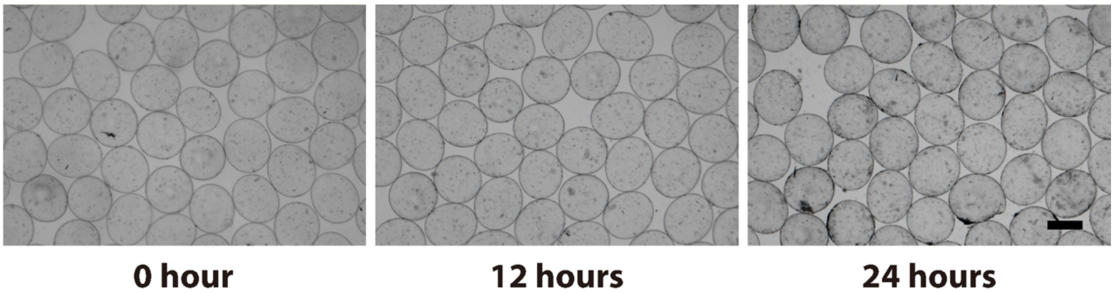

Figure S14. Morphological stability of RGD-Alg@MSCs during storage. Representative light microscopy images of RGD-Alg@MSCs stored at 4°C in PBS for 0, 12, and 24 hours. Scale bar: 100 μm.

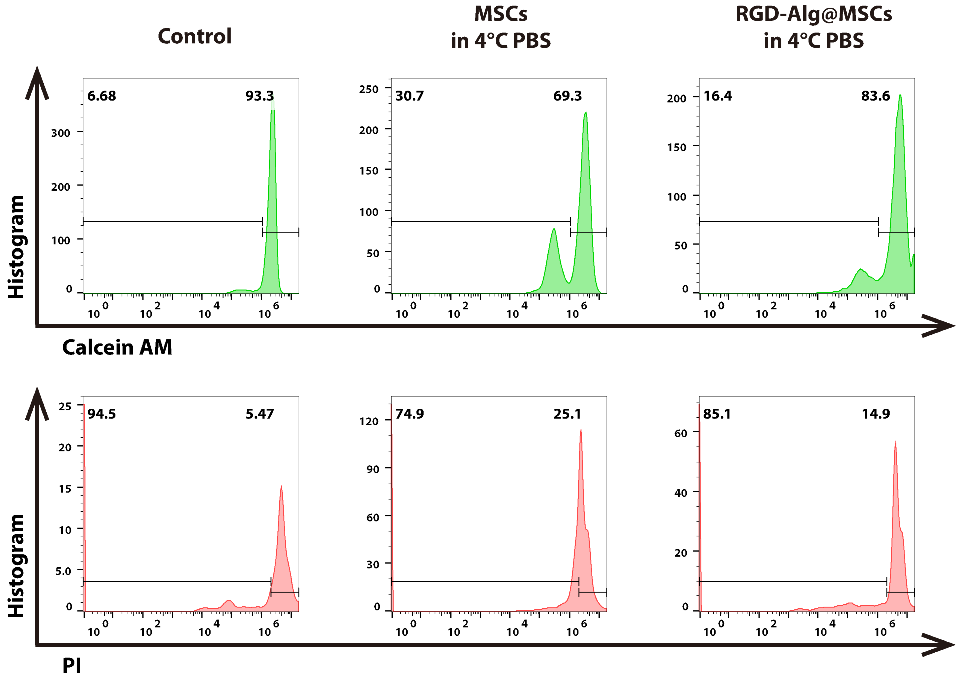

Figure S15. Cell viability analysis of stored MSCs. Flow cytometry results showing Calcein AM/PI staining of MSCs under different storage conditions after 24 hours.

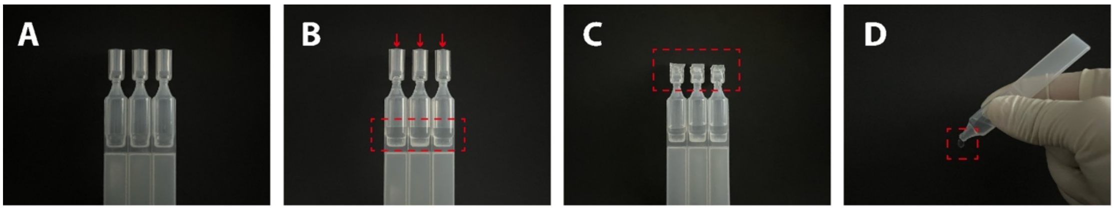

Figure S16. Clinical application protocol of RGD-Alg@MSCs eye drops. (A) Sterile packaging in pharmaceutical-grade dropper bottles. (B) Aseptic filling under sterile conditions. The red arrows indicate the filling point and the red dashed box highlights the filled solution. (C) Cold storage at 4°C with proper sealing. The red dashed box highlights the sealed area. (D) Patient application showing shake-before-use and topical administration. The red dashed box highlights the RGD-

Alg@MSCs eye drops.

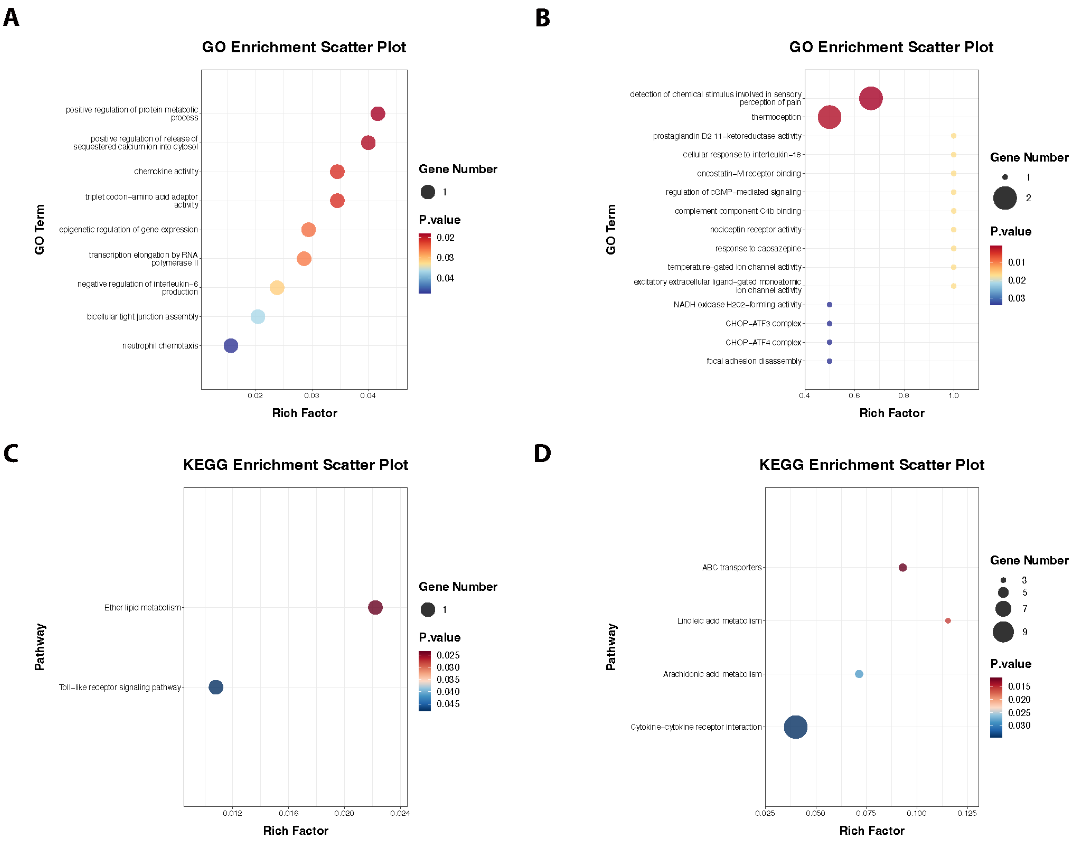

Figure S17. RNA sequencing and bioinformatics analysis of HCECs under hyperosmolarity co-cultured with MSCs or RGD-Alg@MSCs compared to the untreated group. GO enrichment scatter plots of differentially expressed genes (DEGs) in HCECs under hyperosmolar conditions: (A) RGD-Alg@MSCs-Treated vs. Untreated and (B) MSCs-Treated vs. Untreated. KEGG enrichment scatter plots of pathways in HCECs under hyperosmolar conditions: (C) RGD-Alg@MSCs-Treated vs. Untreated and (D) MSCs-Treated vs. Untreated. Untreated refers to HCECs exposed to hyperosmolar conditions without any treatment.

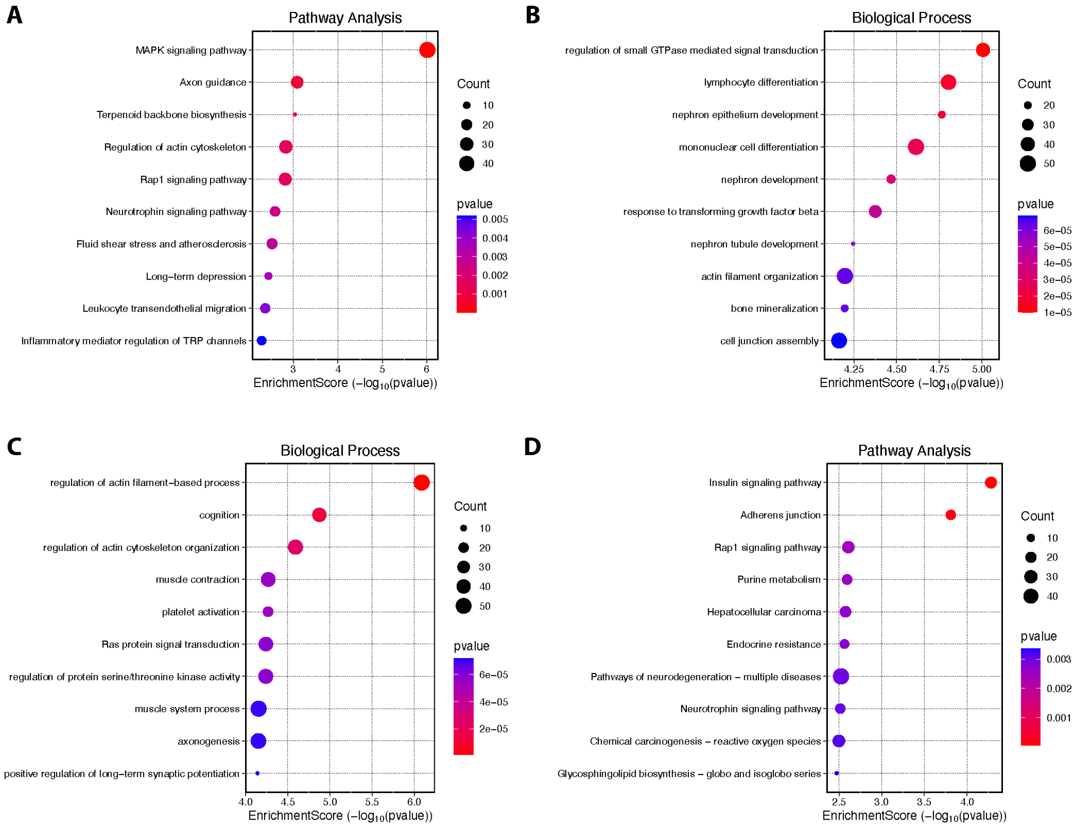

Figure S18. RNA sequencing and bioinformatics analysis of RGD-Alg@MSCs and MSCs compared to the control group. GO enrichment bubble plots of differentially expressed genes (DEGs) for (A) RGD-Alg@MSCs vs. Control and (B) MSCs vs. Control. KEGG pathway enrichment bubble plots for (C) RGD-Alg@MSCs vs. Control and (D) MSCs vs. Control. Control represents MSCs grown in 2D conditions without co-culturing with HCECs.

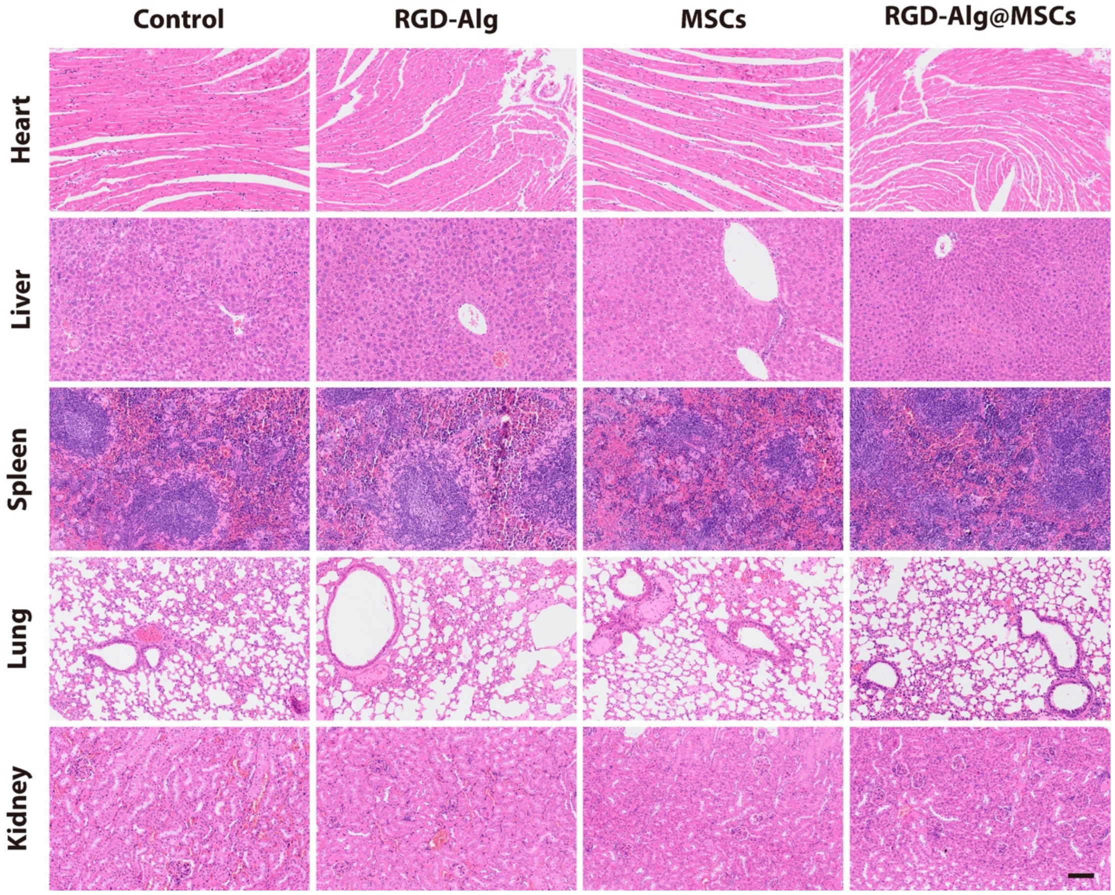

Figure S19. An illustration of the main organs stained with HE. 100 μm is the scale bar.

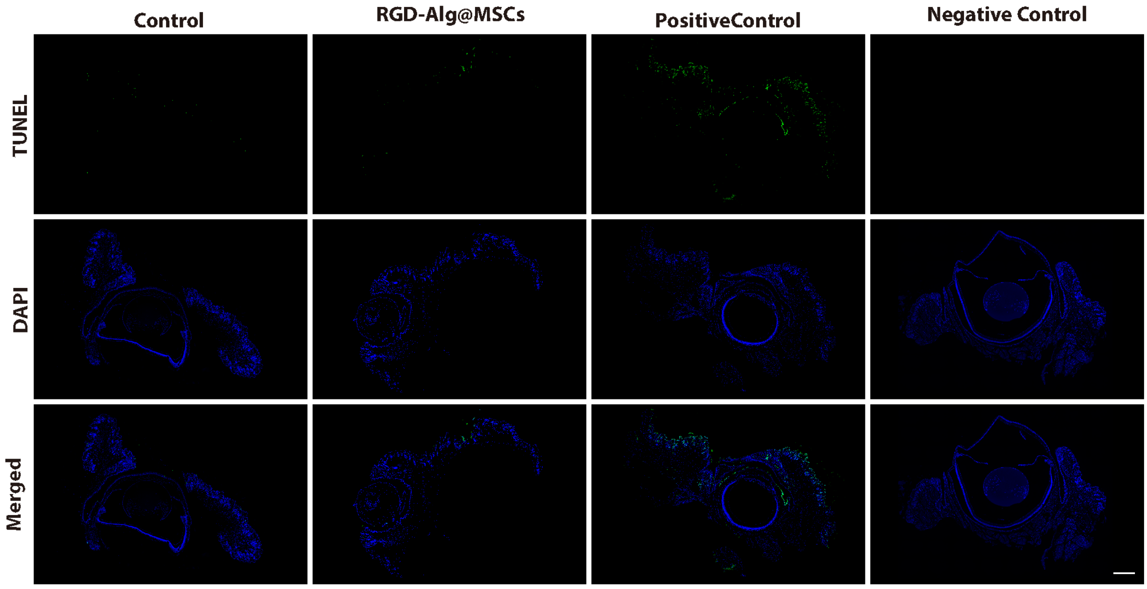

Figure S20. TUNEL staining of eyeballs. The scale bar is 500 μm.

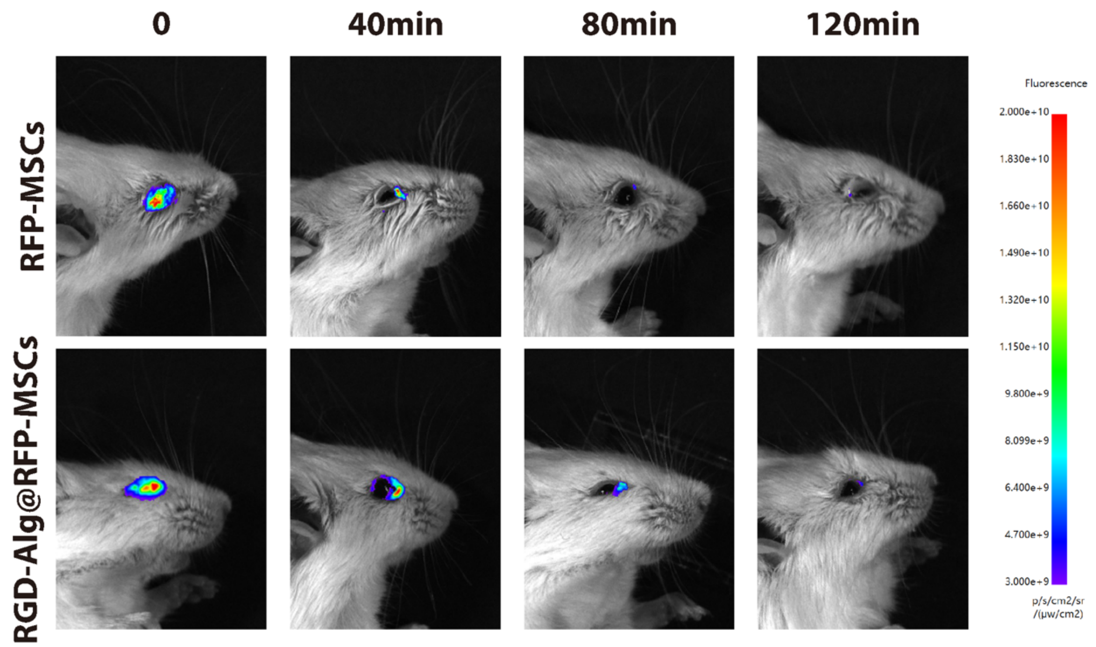

Figure S21. Representative fluorescence images showing the retention time of RFP-MSCs at the ocular surface *in vivo*.

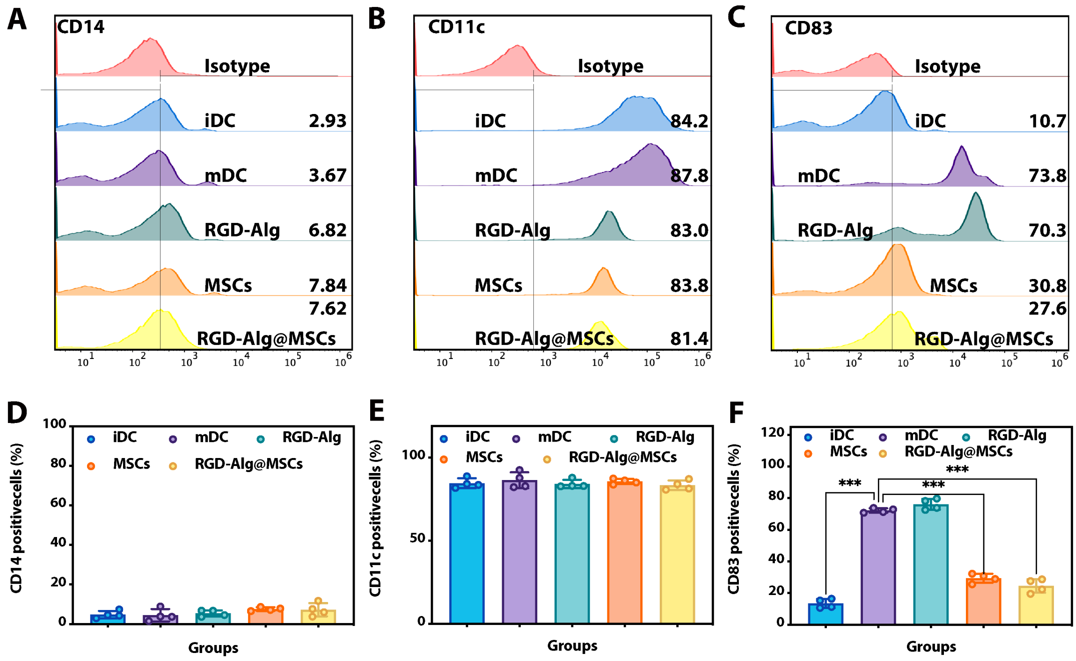

Figure S22. Flow cytometric analysis and quantification of typical cell surface markers in DCs. Flow cytometric analysis showing the expression of representative cell surface markers CD14 (A), CD11c (B) and CD83 (C) of mDCs in different co-culture treatment groups. The uninduced iDCs group was used as the control. Quantification of the percentage of cells positively expressing these

cell surface markers (D,E,F, n = 4). mDCs : mature dendritic cells; iDCs : immature dendritic cells; Isotype: isotype control.

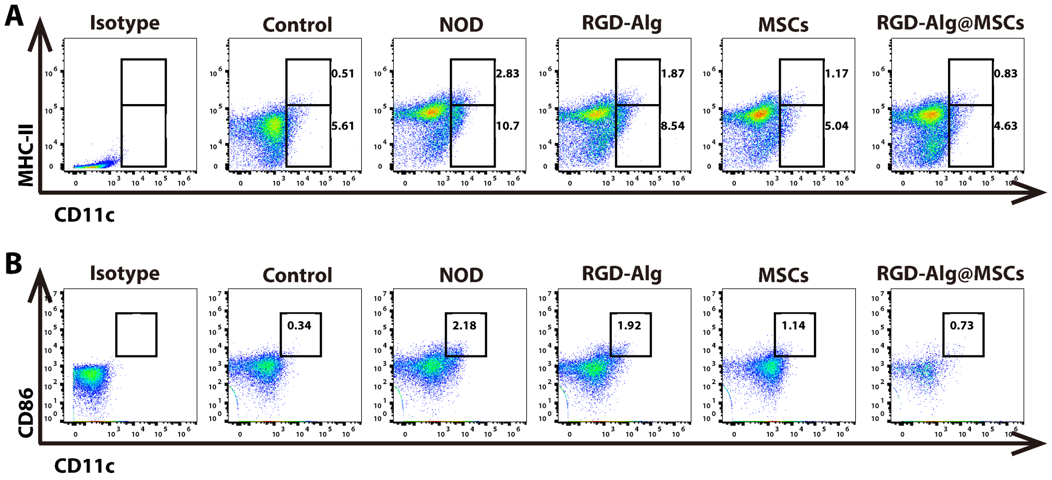

Figure S23. Flow cytometric analysis of DCs. Flow cytometric test for the number (A) and maturity (B) of DCs in the drainage lymph nodes of NOD mice.

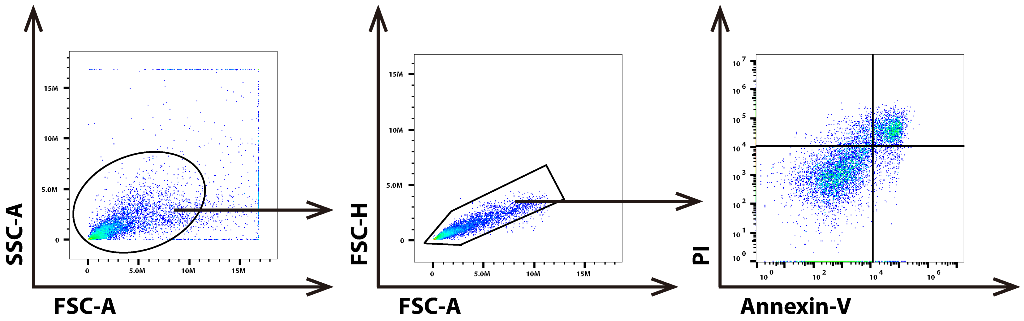

Figure S24. Using flow cytometry, the apoptosis rate in HCECs across groups was evaluated. The gating rule for the flow cytometry test is shown in the figure. The gating strategies are justified by single positive control. A representative sample from the HCEC apoptosis experiment (related to Figure 3D) is used here to illustrate the gating strategy.

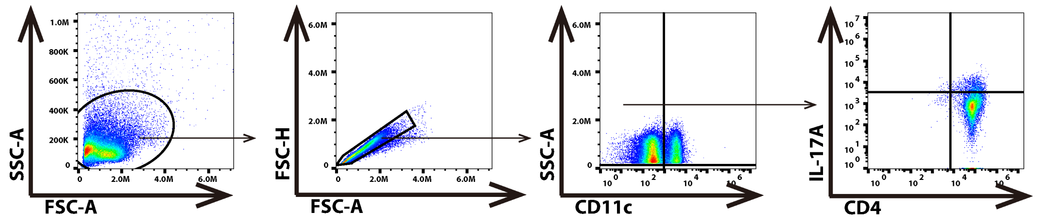

Figure S25. DCs treated with different methods were co-cultured with naïve CD4 + T cells isolated from PBMCs , and flow cytometry was used to detect the gating strategy for the proportion of Th17 cells (CD11c - CD4 + IL-17A +) in them. The plot shown here is identical to the representative data for the mDC group in Figure 6G, and serves to illustrate the gating strategy applied for that analysis.

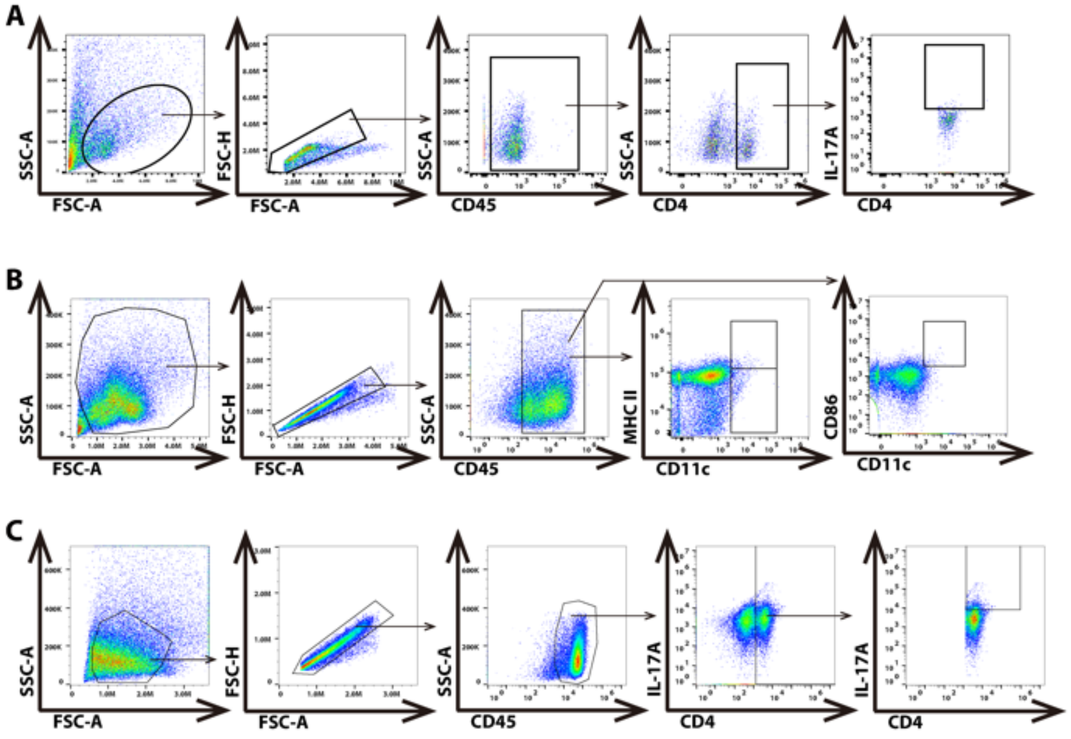

Figure S26. Single-cell preparations from the cornea and draining lymph nodes (dLNs) were made and subjected to flow cytometry analysis in order to identify the impact on NOD mice. The picture illustrates the gating rule for the flow cytometry test for Th17 cells in the cornea (A), DCs (B), and Th17 cells (C) in the dLNs. Appropriate isotype control antibodies and single-stained

controls support the gating techniques. Panel (A) is identical to the representative plot for the RGD-Alg group in Figure 7B and serves to demonstrate the gating strategy for that analysis. Panels (B) and (C) show representative gating strategies using samples from the same experimental batches analyzed in Figure S23 and Figure 7F, respectively.

Table S1. List of individual gene primers.

Gene	NCBI Gene ID	Forward	Reverse
hTNF-α	7124	CCTCCCTAATCAGCCCTCTG	GAGGACCTGGGAGTAGATGAG
hIL-6	3569	GACAGCCACTCACCTCTTCA	CCTCTTTGCTGCTTTCACAC
hIL-1β	3553	CGAATCTCCGACCACCACTA	AGGGAAAGAAGGTGCTCAGG
hβ-Actin	60	GGCACCCAGCACAATGAAG	CCGATCCACACGGAGTACTTG
mTNF-α	20698	AGGCACTCCCCCAAAAGATG	CTTGGTGGTTTGCTACGACG
mIL-1β	16176	TGTGAAATGCCACCTTTTGA	GGTCAAAGGTTTGGAAGCAG
mIL-6	16193	GATGGATGCTACCAAACTGGA	TCTGAAGGACTCTGGCTTTG
mβ-Actin	11461	TCATCACTATTGGCAACGAGC	AACAGTCCGCCTAGAAGCAC

Table S2. List of ELISA.

Protein	Reactivity	Brand name	Cat number
TSG-6	Human	JINGMEI Biotechnology	JM-1217
CCL-20	Human	Solarbio	SEKH-0249
IDO	Human	Solarbio	SEKH-0502
PGE2	Human	Solarbio	SEKSM-0034
